# Association of PTPN22 1858C/T Polymorphism with Autoimmune Diseases: A Systematic Review and Bayesian Approach

**DOI:** 10.3390/jcm8030347

**Published:** 2019-03-12

**Authors:** Kalthoum Tizaoui, Seon Hui Kim, Gwang Hun Jeong, Andreas Kronbichler, Kwang Seob Lee, Keum Hwa Lee, Jae Il Shin

**Affiliations:** 1 Department of Basic Sciences, Division of Histology and Immunology, Faculty of Medicine Tunis, Tunis El Manar University, Tunis 1068, Tunisia; kalttizaoui@gmail.com; 2 School of Medicine, Kyungpook National University, Daegu 41944, Korea; poom528@gmail.com; 3 College of Medicine, Gyeongsang National University, Jinju 52727, Korea; pearlmed15@gmail.com; 4 Department of Internal Medicine IV (Nephrology and Hypertension), Medical University Innsbruck, 6020 Innsbruck, Austria; andreas.kronbichler@i-med.ac.at; 5 Severance Hospital, Yonsei University College of Medicine, Seoul 03722, Korea; KWANGSEOB@yuhs.ac; 6 Department of Pediatrics, Yonsei University College of Medicine, Yonsei-ro 50, Seodaemun-gu, C.P.O. Box 8044, Seoul 03722, Korea; AZSAGM@yuhs.ac; 7 Department of Pediatric Nephrology, Severance Children’s Hospital, Seoul 03722, Korea; 8 Institute of Kidney Disease Research, Yonsei University College of Medicine, Seoul 03722, Korea

**Keywords:** autoimmune disease, single nucleotide polymorphism, PTPN22, false-positive report probability, Bayesian false discovery probability, genome wide association study, meta-analysis

## Abstract

The 1858T allele in the protein tyrosine phosphatase non-receptor type 22 (PTPN22) locus shows one of the strongest and most consistent genetic associations with autoimmune diseases. We synthesized all meta-analyses reporting a genetic association of the PTPN22 1858T C/T polymorphism with autoimmune diseases. This work examined their validity to discover false positive results under Bayesian methods. We conducted a PubMed search to identify relevant publications and extracted the respective results, published until 30 November 2018. In observational studies, the associations of 1858 C/T genetic variant were noteworthy for 12 autoimmune or autoimmunity-related diseases (rheumatoid arthritis, systemic lupus erythematosus, type 1 diabetes mellitus, juvenile idiopathic arthritis, Crohn’s disease, anti-neutrophil cytoplasmic antibody (ANCA)-associated vasculitis, vitiligo, Graves’ disease, myasthenia gravis, Addison’s disease, giant cell arteritis, and endometriosis). In contrast, we could not confirm the noteworthiness for eight diseases (systemic sclerosis, psoriasis, Behçet’s disease, autoimmune thyroid disease, alopecia areata, Sjögren’s syndrome, inflammatory bowel disease, and ankylosing spondylitis). From the meta-analysis of genome-wide association studies (GWAS) with a *p*-value < 5 × 10^−8^, findings verified noteworthiness for all autoimmune diseases (psoriatic arthritis, myasthenia gravis, juvenile idiopathic arthritis and rheumatoid arthritis). The results from meta-analysis of GWAS showing a *p*-value ranging between 0.05 and 5 × 10^−8^ were noteworthy under both Bayesian approaches (ANCA-associated vasculitis, type 1 diabetes mellitus, giant cell arteritis and juvenile idiopathic arthritis). Re-analysis of observational studies and GWAS by Bayesian approaches revealed the noteworthiness of all significant associations observed by GWAS, but noteworthiness could not be confirmed for all associations found in observational studies.

## 1. Introduction

The 1858T allele in the protein tyrosine phosphatase non-receptor type 22 (PTPN22) locus has a strong and consistent genetic association with autoimmune diseases. This phosphatase is expressed in hematopoietic cells and in immune cells with highest levels found in neutrophils and natural killer cells [1]. The PTPN22 gene is located on chromosome 1p 13.3–13.1 and encodes the cytoplasmic lymphoid specific phosphatase (Lyp) [2]. 

Many single nucleotide polymorphisms (SNPs) have been identified in PTPN22, but only one non-synonymous SNP has been intensively studied in relation to autoimmune diseases. The SNP rs2476601 is a change of cytosine to thymidine at nucleotide 1858 (C1858T) which results in an amino acid change from arginine to tryptophan at codon 620 (R620W). This codon is located in the polyproline binding motif P1 [2,3]. The amino acid substitution is located in the polyproline motif within the Lyp protein and, thus, is thought to be involved in binding to SH3 domains during protein–protein interactions [4]. Lyp interacts with C-terminal Src kinase (Csk) to regulate both B-cell receptor (BCR) and T-cell receptor (TCR) signaling [5,6]. It has been suggested that 1858 C/T polymorphism increases Lyp protein activity resulting in inhibition of T-cell signaling and a failure to delete autoreactive T-cells during thymic selection. Since 1858 C/T polymorphism results in immune responses against autoantigens [3], genetic association is proposed to be restricted to disorders that have a strong autoantibody component. 

There is no consensus whether 1858 C/T polymorphism is a gain- or loss-of-function variant. The C1858T has been reported as a susceptibility locus associated with several autoimmune diseases. It was first reported in type 1 diabetes mellitus (T1DM) [2]. Numerous studies have confirmed this association [7,8], and implication of the PTPN22 1858 C/T polymorphism was also proposed in other autoimmune diseases [8]. While implicated in the genetic basis of autoimmunity, the 1858 C/T polymorphism may protect individuals from environmental pathogens [9]. 

In recent years, the advent of new genotyping and other molecular biology technologies has provided a huge increase in the quantities of data available for analysis. In epidemiology, candidate-gene and genome-wide association identified a large number of genes associated with diseases. Therefore, a general focus has been laid on the way genetic associations are reported. Meta-analysis has been widely used as a powerful approach to identify true-positive associated genes, but several limitations can alter the results. In most cases, results between overlapping meta-analyses on the same topic are inconsistent because of several confounding factors such as inclusion and exclusion criteria and number of included studies. 

In addition, since the prior probabilities of genetic associations are low, there is a possibility that the number of false-positive associations by chance may be high, which could lead to an increased likelihood of finding false-positive associations. In the present study, we used Bayesian approaches [10,11,12] because they represent a more powerful tool than other methods for detecting true noteworthiness (true associations) for the genetic associations between the gene variant and disease. Bayesian approaches depend not only on the observed P value but also on both the prior probability that the association between the genetic variant and the disease is real (genuine) and the statistical power of the test [10]. Although a strict *p*-value of <5 × 10^−8^ was set to determine the statistical significance in genome-wide association studies (GWAS) or its meta-analysis, a significance level of observational studies on genetic epidemiology (*p*-value of <5 × 10^−8^) has not been changed. Therefore, judging the true significance between the suggested gene variant and disease is very important and Bayesian approaches can detect the claimed associations are genuine (true or false), which would be the reason why noteworthiness is so important. Bayesian approaches allow researchers to consider a much broader class of conceptual and mathematical models and permit to work on complex analytical problems, irrespective of the size of data [10,11,12]. 

In this review, we have synthesized all available data reporting the association of the PTPN22 1858 C/T polymorphism with autoimmunity retrieved from meta-analyses. Both meta-analyses of observational studies and GWAS were included and Bayesian approaches have been employed to estimate the noteworthiness of the evidence. We aimed to provide an overview to interpret the reported significant findings and discuss the genetic association of autoimmunity with the PTPN22 1858 C/T polymorphism.

## 2. Methods

### 2.1. Inclusion and Exclusion Criteria

Studies were included if they satisfied the following criteria: (1) estimated the risk conferred by PTPN22 in autoimmune diseases using meta-analyses which reported odds ratio (OR) and 95% confidence interval (CI), and (2) published in English.

Articles were excluded if (1) they did not report PTPN22 1858C/T polymorphism or autoimmune or autoimmunity-related diseases, (2) did not perform meta-analysis and (3) the original study did not report upper and lower confidence intervals, because these are necessary to calculate false-positive report probability (FPRP) and Bayesian false discovery probability (BFDP) (1 from observational studies and 7 from meta-analyses of GWAS, Supplementary Tables S1 and S2). The eligible studies were selected according to the standardized reporting protocol of systematic reviews and meta-analyses PRISMA (Preferred Reporting Items for Systematic reviews and Meta-Analyses) checklist (Supplementary Table S3).

### 2.2. Search Strategy

**Figure 1:**
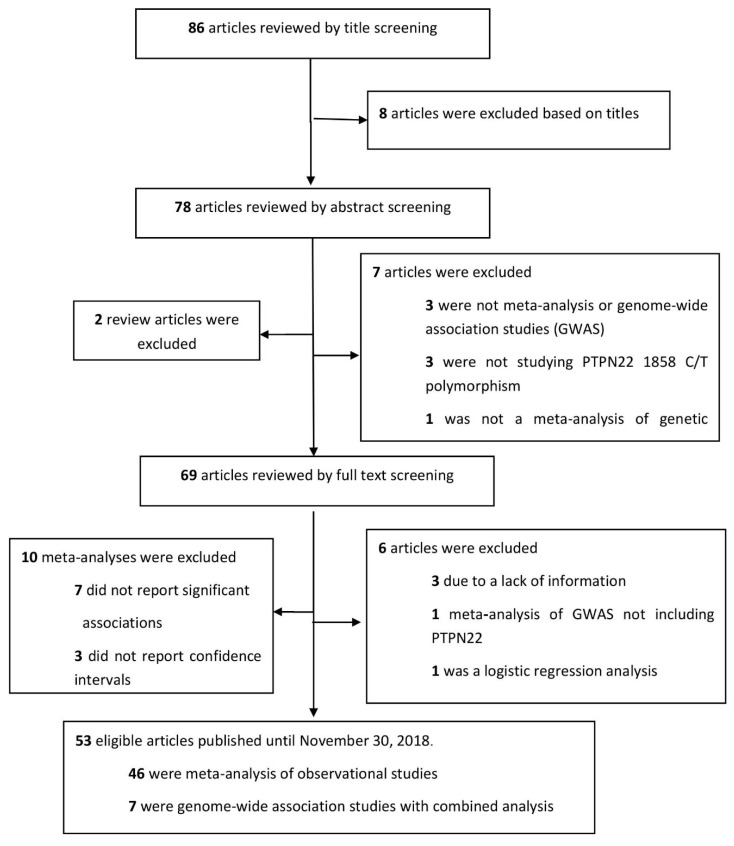
The process of the systematic search performed to study the PTPN22 polymorphism in autoimmune or autoimmunity-related diseases.

A PubMed search was performed to extract data from meta-analyses regarding the PTPN22 1858C/T polymorphism in autoimmune or autoimmunity-related diseases published until 30 November 2018. We used the search terms “PTPN22” AND “polymorphism” AND “meta”. Out of 86 yielded articles, 17 articles were excluded after screening the title and abstract. Full text screening led to the exclusion of another 6 articles. Additional 10 meta-analyses were excluded because they did not report significant associations or lower and upper limits of the confidence interval. From a total of 53 eligible studies, 46 were meta-analyses of observational studies and 7 studies were meta-analyses of GWAS [Supplemental references] (Figure 1).

### 2.3. Data Extraction

We classified 53 articles into two categories depending on the methods of the respective studies, either meta-analysis of observational studies or GWAS. Data were extracted from each article, including the type of autoimmune disease, clinical symptoms, genetic variant, genotype comparison, odds ratio (OR), 95% confidence interval (CI), *p*-value, statistical model used for analysis (i.e., either random or fixed), ethnicity of participants, the number of cases and controls, heterogeneity described with I^2^ or *p*-value and publication bias assessed as Egger’s *p*-value [13].

The reported associations were significant if the *p*-value was lower than 0.05, equally described as 95% CI excluding 1.0 in meta-analyses of observational studies (Table 1, Table 2, Table 3, Table 4, Table 5, Table 6 and Table 7) and 5 × 10^−8^ in meta-analyses of the GWAS (Table 8). Genotypic and allelic comparisons from GWAS with a *p*-value between 0.05 and 5 × 10^−8^ had a borderline significance and were separately organized in Table 9.

### 2.4. Statistical Analysis

To verify the noteworthiness of the reported genetic association, we used the Bayesian approaches, FPRP and BFDP. In our study, noteworthiness was used as a term that a genetic association of PTPN22 is associated with a disease in a significant manner. FPRP, proposed by Wacholder et al., tests the probability of no true association between the polymorphism and the disease [10]. FPRP can be derived from the following equation: FPRP = α (1 − π)/{α (1 − π) + (1 − β)} in which (1) π (the prior probability of a true association), (2) the lowest α where the test is noteworthy or the observed *p*-value, and (3) 1 − β (the statistical power at which the finding is defined as noteworthy) [11].

We used two pre-specified values for prior probabilities (10^−3^ and 10^−6^) and two values of OR (1.2, 1.5) which were thought to be valid for a noteworthy finding were chosen. FPRP was calculated by an Excel spreadsheet reported by Wacholder et al. [10] and a FPRP value less than 0.2 was regarded as noteworthy genetic association.

Secondly, BFDP, newly proposed by Wakefield et al. [12], also clarifies the noteworthiness of the reported genetic association. It provides a description of a noteworthiness by means of the cost of a false discovery and a false non-discovery, while utilizing more information than FPRP [12]. According to the literature, the cutoff level for BFDP is set at 0.8, derived from the assumption that a false non-discovery is four times as costly as a false discovery. BFDP can also be calculated by the following equation and an Excel spreadsheet was reported by Wakefield et al. [12], where PO is the prior odds of no association and ABF is the approximate Bayes factor which can be deduced from OR and SE.

BFDP = (ABF × PO)/(ABF × PO + 1)

We also used two pre-specified values for prior probabilities (10^−3^ and 10^−6^) and a BFDP value less than 0.8 was noteworthy. Each comparison of the genetic associations was regarded as noteworthy when FPRP of <0.2 or BFDP of <0.8 or both were fulfilled. We adopted these cut-offs of FPRP and BFDP, because these were set by the original authors (Wacholder et al. and Wakefield et al., respectively) [10,12].

All the calculations to derive FPRP and BFDP were performed with the Excel spreadsheet released by Wacholder et al. and Wakefield et al. [10,12]. The values for FPRP and BFDP of the observational studies were specified in Table 1, Table 2, Table 3, Table 4, Table 5, Table 6 and Table 7. Results for GWAS were separately described in Table 8 and Table 9 depending on whether *p*-value was adequately significant (*p* < 5 × 10^−8^) or was situated on the boundary of statistical significance (0.05 < *p* < 5 × 10^−8^).

## 3. Results

The genetic association of the PTPN22 1858 C/T variant was evaluated in a total of 20 autoimmune or autoimmunity-related diseases. Most studies focused on rheumatoid arthritis (RA, *n* = 13), systemic lupus erythematosus (SLE, *n* = 7) and type 1 diabetes mellitus (T1DM, *n* = 8), followed by five on juvenile idiopathic arthritis (JIA), 5 on Crohn’s disease (CD), 4 on anti-neutrophil cytoplasmic antibody (ANCA)-associated vasculitis, 3 on vitiligo, 3 on systemic sclerosis (SSc), 3 on Graves’ disease (GD), 3 on myasthenia gravis (MG), 3 on Addison’s disease (AD), 2 on psoriasis, 1 study on Behcet’s disease (BD), 1 on endometriosis, 1 on autoimmune thyroid disease (AITD), 1 on inflammatory bowel disease (IBD), 1 on giant cell arteritis (GCA), 1 on alopecia areata (AA), 1 on Sjögren’s syndrome (SS), and 1 study on ankylosing spondylitis (AS). 

In observational studies, the associations of 1858 C/T genetic variant were noteworthy for 12 autoimmune or autoimmunity-related diseases (RA, T1DM, SLE, JIA, CD, ANCA-associated vasculitis, vitiligo, GD, MG, AD, GCA, and endometriosis). In contrast, the results did not show noteworthiness for eight diseases (SSc, psoriasis, BD, AITD, AA, SS, IBD and AS).

### 3.1. Rheumatoid Arthritis

A total of 13 observational studies with 39 genotypes and allelic comparisons were included. Most studies used the general population as the comparators. Out of 39 RA comparisons, 10 and 4 were verified to be noteworthy (<0.2) using the FPRP estimation, at a prior probability of 10^−3^ and 10^−6^ with a statistical power to detect an OR of 1.2. In addition, 13 and 11 comparisons were verified to be noteworthy at a prior probability of 10^−3^ and 10^−6^ with a statistical power to detect an OR of 1.5. With the use of BFDP, 32 and 30 comparisons showed noteworthiness at a prior probability of 10^−3^ and 10^−6^, respectively. In total, 32 (82.1%) of the 39 comparisons had noteworthy findings by FPRP or BFDP (Table 1).

### 3.2. Juvenile Idiopathic Arthritis

Five studies with 15 genotype and allele comparisons were included. By means of FPRP estimation, 5 and 3 findings were noteworthy at a prior probability of 10^−3^ and 10^−6^ with a statistical power to detect an OR of 1.2, respectively. Moreover, 7 and 4 were noteworthy at a prior probability of 10^−3^ and 10^−6^ with a statistical power to detect an OR of 1.5, respectively. In terms of BFDP estimation, 8 and 6 comparisons showed worthiness at a prior probability of 10^−3^ and 10^−6^, respectively. In total, 9 (60%) of the 15 comparisons had noteworthy findings by FPRP or BFDP (Table 2).

### 3.3. Systemic Lupus Erythematosus

Seven observational studies with 15 genotypes and allelic comparisons were identified. Out of 15 comparisons, 8 and 3 were noteworthy using FPRP estimation, at a prior probability of 10^−3^ and 10^−6^ with a statistical power to detect an OR of 1.2. In addition, 11 and 6 showed noteworthiness at a prior probability of 10^−3^ and 10^−6^ with a statistical power to detect an OR of 1.5. In terms of BFDP, 13 and 8 comparisons had noteworthy findings at a prior probability of 10^−3^ and 10^−6^ (Table 3). In total, 13 (86.7%) of the 15 comparisons had noteworthy findings by FPRP or BFDP.

### 3.4. Vasculitides

A total of 4 studies with 11 genotypes and allelic comparisons were included for ANCA-associated vasculitis. Out of 11 comparisons, 3 were noteworthy using FPRP estimation, at a prior probability of 10^−3^ with a statistical power to detect an OR of 1.2. In addition, 6 and 2 were verified to be noteworthy at a prior probability of 10^−3^ and 10^−6^ with a statistical power to detect an OR of 1.5 by FPRP. In terms of BFDP, 6 and 2 comparisons had noteworthy findings at a prior probability of 10^−3^ and 10^−6^, respectively (Table 4). In total, 6 (54.5%) of the 11 comparisons had noteworthy findings by FPRP or BFDP.

For the studies including subjects with GCA, only one study with three allelic comparisons was included. Two comparisons verified noteworthiness at a prior probability of 10^−3^ with a statistical power to detect an OR of 1.5. By using BFDP, two results were noteworthy at a prior probability of 10^−3^ (Table 4).

### 3.5. Other Rheumatic Autoimmune Diseases

Two studies with three allelic comparisons analyzed the genetic impact of psoriasis and did not verify noteworthiness by means of both FPRP and BFDP estimations (Table 5). Three studies including patients with SSc analyzed 7 genotypes and allelic comparisons. Findings did not show noteworthiness in terms of FPRP and BFDP estimations (Table 5). Two studies examined associations of psoriasis and only one study was available for each SS and AS. Findings from these diseases did not verify noteworthiness by means of FPRP and BFDP estimations (Table 5).

### 3.6. Other Autoimmune or Other Disorders

Three studies with 5 comparisons were included from patients with vitiligo. In terms of FPRP estimation, 4 results were only noteworthy at a prior probability of 10^−3^ with a statistical power to detect an OR of 1.2. In addition, four results were noteworthy at a prior probability of 10^−3^ and 10^−6^ with a statistical power to detect an OR of 1.5. Using BFDP estimation, findings were noteworthy except for generalized vitiligo which did not show any noteworthiness by using both FPRP and BFDP estimations (Table 6). In total, 4 (80%) of the 5 comparisons had noteworthy findings by FPRP or BFDP.

Five studies including patients with CD had five comparisons. In terms of FPRP, two findings were noteworthy at a prior probability of 10^−3^ with a statistical power to detect an OR of 1.2. Moreover, noteworthiness was reported for 2 results and 1 result at a prior probability of 10^−3^ and 10^−6^, respectively, with a statistical power to detect an OR of 1.5, respectively. By using BFDP estimation, only 2 results were noteworthy at a prior probability of 10^−3^ (Table 6). In total, 2 (40%) of the 5 comparisons had noteworthy findings by FPRP or BFDP.

Three studies reporting five allelic comparisons were included from subjects with MG. Out of 5 comparisons, 3 findings were noteworthy, by using FPRP, at a prior probability of 10^−3^ with a statistical power to detect an OR of 1.2. In addition, 4 and 2 verified noteworthiness at a prior probability of 10^−3^ and 10^−6^ with a statistical power to detect an OR of 1.5. In terms of BFDP, 4 and 2 results were noteworthy at a prior probability of 10^−3^ and 10^−6^, respectively (Table 6). In total, 4 (80%) of the 5 comparisons had noteworthy findings by FPRP or BFDP. In addition, there were no noteworthy findings by FPRP or BFDP in one study (2 comparisons) of Behçet’s disease and one study (3 comparisons) of AITD.

Three studies with 3 allelic comparisons were included for Addison’s disease. Out of the three comparisons, 2 were noteworthy at a prior probability of 10^−3^ with a statistical power to detect an OR of 1.5. In terms of BFDP, 2 findings verified noteworthiness at a prior probability of 10^−3^. For patients with endometriosis, one study with three co-dominant comparisons did not verify noteworthiness, except for one finding which was noteworthy by using BFDP at a prior probability of 10^−3^. There were no noteworthy findings by FPRP or BFDP in one study (1 comparison) of alopecia areata (Table 6).

### 3.7. Type 1 Diabetes Mellitus

Regarding the association of PTPN22 and T1DM, 8 studies with 22 comparisons were included in the analysis. Out of 22 comparisons, 4 verified noteworthiness using FPRP estimation, at a prior probability of 10^−3^ with a statistical power to detect an OR of 1.2. In addition, 7 and 3 comparisons were noteworthy at a prior probability of 10^−3^ and 10^−6^ with a statistical power to detect an OR of 1.5, respectively. By using BFDP estimation, 19 and 14 findings were noteworthy at a prior probability of 10^−3^ and 10^−6^, respectively (Table 7). In total, 19 (86.4%) of the 22 comparisons had noteworthy findings by FPRP or BFDP.

### 3.8. Meta-Analysis of Genome-Wide Association Studies

Among the included GWAS meta-analyses, findings verified noteworthiness for all included diseases. Four studies with a *p*-value <5 × 10^−8^ showed noteworthiness by FPRP or BFDP for PsA, MG, RA and JIA. All comparisons were based on Caucasian populations. Out of four genotype and allelic comparisons, three were verified to be noteworthy (<0.2) using FPRP estimation, at a prior probability of 10^−6^ with a statistical power to detect an OR of 1.2 and 1.5. By means of BFDP, the four comparisons had noteworthy findings (<0.8) at a prior probability of 10^−3^ and 10^−6^ (Table 8). In four studies with a non-significant GWAS *p*-value (5 × 10^−8^ < *p* < 0.05), findings were noteworthy for ANCA-associated vasculitis, T1DM, GCA and JIA. The four comparisons were verified to be noteworthy using FPRP estimation, at a prior probability of 10^−6^ with a statistical power to detect an OR of 1.2 and 1.5. In terms of BFDP, results were noteworthy at a prior probability of 10^−3^ (Table 9).

In addition, we were unable to estimate noteworthiness of 32 potential associations under FPRP due to a mathematical error while performing a calculation using excel. It was considered that a substantially low *p*-value with a narrow CI hindered the computation for obtaining the inverse of the cumulative normal distribution. 

## 4. Discussion

The current work is a comprehensive search of the literature which outnumbers previous meta-analyses focusing on the association of PTPN22 1858 C/T polymorphism and autoimmune diseases and is the first work applying Bayesian procedures such as FPRP and BFDP to prove the noteworthiness of such associations.

We describe the results of genotype associations that were found to be noteworthy through FPRP and BFDP estimations and these Bayesian statistical methods were useful to detect true noteworthiness (genuine associations) for the genetic associations between the gene variant and disease. In recent years, Bayesian methods have been increasingly used because of their extreme flexibility as a major advantage. Bayesian methods can provide researchers with gains in performance of statistical estimation by incorporating prior information. BFDP allows the calculation of the recently proposed FPRP but uses more information [10,12]. These methods were introduced as criteria, “to help investigators, editors, and readers of research articles to protect themselves from over interpreting statistically significant findings that are not likely to signify a true association” [12].

In observational studies, the associations of the 1858 C/T genetic variant were noteworthy for 12 autoimmune or autoimmunity-related diseases (RA, T1DM, SLE, JIA, CD, ANCA-associated vasculitis, vitiligo, GD, MG, AD, GCA, and endometriosis) and the positive rate of true noteworthiness was different among diseases. The role of the pleiotropic 1858C/T of PTPN22 may suggest common and shared immune functions in various autoimmune diseases. More functional studies in the future may identify specific effects of this polymorphism in each autoimmune disease.

However, the results did not show noteworthiness for the remainder, highlighting the need for further investigations. The non-significant association may implicate that the functional effect of C1858T on the Lyp protein is not a major contributing factor to study these autoimmune diseases or that the pathogenic inflammatory responses are not influenced or regulated by this pathway. In addition, the non-synonymous C1858T genetic variant is the only significant SNP out of many within the PTPN22 region, but we suggest that some other synonymous variants in these pathologies should be studied more intensively to elucidate their function. Furthermore, genetic association studies should be replicated in different populations with larger sizes.

All GWAS findings verified noteworthiness for all included diseases. Four studies with a *p*-value <5 × 10^−8^ showed noteworthiness for PsA, MG, RA and JIA. Noteworthiness showing an association of four autoimmune diseases, namely ANCA-associated vasculitis, T1DM, GCA and JIA, could be observed in the GWAS non-significant findings when a *p*-value ranged between 5 × 10^−8^ and 0.05. It can be assumed that GWAS produces a more solid evidence than observational studies with a lower amount of false positive results, because of its stringent threshold to determine a significance. However, other genetic variants had a significant *p*-value (i.e., *p* < 0.05 for observational studies and *p* < 5 × 10^−8^ for GWAS) and found noteworthy by our Bayesian approaches and thus it may be concluded that results from GWAS with a *p*-value < 5 × 10^−8^could be identically replicated in observational studies. Some comparisons that could not be calculated using FPRP (either due to missing reports of upper and lower confidence intervals or a mathematical error in the process of calculating the inverse of the cumulative normal distribution) could be computed using BFDP and were found to be noteworthy in our final analysis.

PTPN22 encodes a protein tyrosine phosphatase that inhibits antigen-receptor signaling in T cells and promotes pattern-recognition receptor-induced type I interferon production by myeloid cells. Zheng et al. [14] proposed that PTPN22 has stronger associations with autoimmune disorders in which auto-antibodies have a major role in pathogenesis. The effect of PTPN22 depends on the respective tissue affected by autoimmunity [14]. Autoimmune diseases affecting connective tissues, joints, muscles, blood, pancreas, kidney or thyroid show a stronger association with PTPN22 than diseases of the gastrointestinal tract or immune-privileged sites, such as the central nervous system and the eye [14]. Genetic mutation plays an important role in the development of autoimmune disease. The PTPN22 1858T variant was among the first SNPs to be associated with multiple autoimmune diseases. Autoimmunity, promoted by PTPN22 1858C/T, involves the differentiation of T-cell subsets, the B-cell repertoire and balance between immunoregulatory and proinflammatory cytokine production [15]. Current studies highlight a role of 1858 C/T polymorphism in autoimmunity by altering innate and adaptive immune responses. Thus, studies of human cells demonstrate the impact of the1858 C/T polymorphism on both maturation and function of hematopoietic lineages, each potentially contributing to autoimmunity [16]. In human lymphocytes, the SNP disrupts the interaction between PTPN22 and Csk [2,17]. The interaction with Csk modulates the inhibitory function of PTPN22 in TCR signaling [17,18]. Considering that some of the strongest associations of the PTPN22 1858C/T are with autoimmune diseases characterized by the production of circulating autoantibodies, dysregulation of B-cell clonal deletion and receptor editing is likely to contribute to PTPN22-associated autoimmune diseases [19]. Previous studies demonstrated a decrease in IL-2 production after TCR stimulation in patients with T1DM carrying the PTPN22 1858T variant [20]. Analysis of individuals with the variant allele and ANCA-associated vasculitis showed a decrease in IL-10 production, which is known to exhibit anti-inflammatory properties [21]. The 1858 C/T polymorphism has also been shown to impair production of type I interferons by myeloid cells [22]. Other SNPs of PTPN22 have been associated with connective tissue diseases. PTPN22 788G>A is a rare missense SNP that does not show co-occurrence with PTPN22 1858C>T [23]. PTPN22 788G>A encodes a loss-of-function Arg263Gln substitution in the PTPN22 catalytic domain, which changes the conformation of the active site and reduces the phosphatase activity of the protein [23]. It has been suggested that the 1858C/T polymorphism is selected in co-evolution with the increase of autoimmune diseases in modern societies. Therefore, the 1858C/T polymorphism is variable in allele frequency across different ethnic groups. There is a noticeable decrease in minor T allele frequencies in Caucasians from northern Europe to southern Europe [24,25]. The highest minor allele frequencies have been reported from Scandinavian countries [26,27], and the lowest minor allele frequency has been observed in Italy [2]. Minor allele frequency of the T allele in US Caucasians ranges from 7% to 9% [28]. In non-Caucasian populations, the 1858 C/T polymorphism is substantially less polymorphic. In fact, this polymorphism has not been found in African or Asian populations [29,30].

Although our report is the most updated and most analytical summary of available evidences on 1858 C/T polymorphism in autoimmune diseases, the review has some limitations. First, it should be noted that the lack of association might reflect the limited power of the studies including only a small number of patients with rare diseases. Secondly, we only included a single result of meta-analysis with the lowest *p*-value per disease. Therefore, we could not consider other factors such as statistical models (i.e., random or fixed), ethnicity, and type of genotype comparison (i.e., recessive, dominant, additive, co-dominant). In addition, despite our efforts, (1) some relevant articles may not have been included based on our search criteria as we considered publications limited to the PubMed database only; (2) some observational studies have been used for more than one meta-analysis, which raises the statistical issue of type 1 error inflation; and (3) we have not considered papers dealing with various permutations and interactions with other genes. Moreover, very few observational studies and GWAS involved African, Middle Eastern, and Asian populations and a population stratification analysis based on the same ethnic and geographic population may require further investigation.

In addition, FPRP often generates much smaller posterior null estimates than BFPD, because FPRP is a lower bound on the posterior probability relevant to the observed estimates [10,12]. However, both approaches may be a beneficial method to distinguish whether the reported associations were genuine or not, especially for interpreting the retrieved results from observational studies, as shown in other previous reports [31,32,33,34,35].

## 5. Conclusions

We attempted to synthesize all meta-analyses on genetic associations of the PTPN22 1858 C>T polymorphism with autoimmune diseases and investigated their validity to discover false positive results under Bayesian methods. To verify results obtained from genetic analyses, both approaches may have advantages, and we were able to confirm significance in almost all autoimmune diseases within this borderline significance range. Therefore, PTPN22 is further confirmed as a candidate gene for further studies. Its promiscuous association with multiple autoimmune diseases might indicate a common mechanism underlying the development of autoimmune disease. Such a finding would have huge implications in our current understanding of autoimmunity and may be of therapeutic benefit and aid in devising preventative strategies. In addition, further studies should be performed to elucidate how PTPN22 1858 C/T could influence the pathogenesis of each autoimmune disease.

## Figures and Tables

**Table 1:** Meta-analysis results of associations between rheumatoid arthritis (RA) and the PTPN22 1858 C/T polymorphism from observational studies.

Author, Year	No. of Studies	Comparison	OR (95% CI)	*p*-Value	Model	Disease	Ethnicity	I^2^ (%)	Egger’s *p*-Value	Power OR 1.2	Power OR 1.5	FPRP Values at Prior Probability				BFDP 0.001	BFDP 0.000001
												OR 1.2		OR 1.5			
												0.001	0.000001	0.001	0.000001		
Nabi G, 2016 [1S]	35	TT vs. CC+CT	2.6 (2.273–3.089)	**<0.0001**	F	RA	Caucasian	0	>0.05	NA	NA	NA	NA	NA	NA	**0.000**	**0.000**
Nabi G, 2016 [1S]	8	T vs. C	1.22 (0.99–1.496)	**0.061**	F	RA	Asian	31.16	>0.05	0.447	0.976	0.993	1.000	0.985	1.000	0.998	1.000
Elshazli R, 2015 [2S]	29	CT+TT vs. CC	1.79 (1.604–2.006)	**<0.001**	R	RA	Overall mixed	60.34	0.038	0.000	0.017	**0.000**	**0.065**	**0.000**	**0.000**	**0.000**	**0.000**
Elshazli R, 2015 [2S]	18	CT+TT vs. CC	1.68 (1.579–1.793)	**<0.001**	F	RA	European	14.05	0.141	NA	NA	NA	NA	NA	NA	**0.000**	**0.000**
Elshazli R, 2015 [2S]	3	T vs. C	3.68 (1.020–13.312)	**0.047**	R	RA	African	86.51	0.627	0.006	0.085	0.999	1.000	0.998	1.000	0.999	1.000
Elshazli R, 2015 [2S]	2	T vs. C	3.57 (1.534–8.323)	**0.003**	F	RA	Asian	7.10	-	0.006	0.022	0.998	1.000	0.993	1.000	0.998	1.000
Tang GP, 2014 [3S]	32	T vs. C	1.61 (1.518–1.69)	**<0.001**	NA	RA	Overall mixed	NA	-	NA	NA	NA	NA	NA	NA	**0.000**	**0.000**
Tang GP, 2014 [3S]	-	T vs. C	1.612 (1.54–1.68)	**<0.001**	NA	RA	Caucasian	NA	-	NA	NA	NA	NA	NA	NA	**0.000**	**0.000**
Song GG, 2013 [4S]	30	T vs. C	1.49 (1.332–1.668)	**<1.0 × 10^−9^**	R	RA	Overall mixed	71.9	-	0.000	0.546	**0.000**	**0.048**	**0.000**	**0.000**	**0.000**	**0.001**
Song GG, 2013 [4S]	24	T vs. C	1.423 (1.260–1.605)	**1.0 × 10^−8^**	R	RA	European	72.6	-	0.003	0.805	**0.003**	0.770	**0.000**	**0.011**	**0.001**	**0.477**
Song GG, 2013 [4S]	7	T vs. C	1.561 (1.373–1.775)	**<1.0 × 10^−9^**	F	RA (RF+ vs. RF−)	Overall mixed	34.6	-	0.000	0.272	**0.000**	0.266	**0.000**	**0.000**	**0.000**	**0.003**
Song GG, 2013 [4S]	6	CT+TT vs. CC	2.02 (1.721–2.371)	**<1.0 × 10^−9^**	F	RA	Non-European	44.9	-	NA	NA	NA	NA	NA	NA	**0.000**	**0.000**
Zheng J, 2012 [5S]	36	T vs. C	1.65 (1.58–1.71)	**<1.0 × 10^−16^**	-	RA	Overall mixed	-	-	NA	NA	NA	NA	NA	NA	**0.000**	**0.000**
Zheng J, 2012 [5S]	11	T vs. C	1.63 (0.51–1.75)	**<1.0 × 10^−16^**	-	RF+	Overall mixed	-	-	NA	NA	NA	NA	NA	NA	**0.000**	**0.000**
Zheng J, 2012 [5S]	11	T vs. C	1.35 (1.22–1.49)	**9.04 × 10^−9^**	-	RF-	Overall mixed	-	-	0.010	0.982	**0.000**	0.206	**0.000**	**0.003**	**0.000**	**0.180**
Zheng J, 2012 [5S]	11	T vs. C	1.78 (1.59–2.01)	**<1.0 × 10^−16^**	-	RA (anti-CCP+)	Overall mixed	-	-	NA	NA	NA	NA	NA	NA	**0.000**	**0.000**
Lee YH, 2012 [6S]	18	T vs. C	1.64 (1.51–1.77)	**<0.001**	R	RA	Overall mixed	32.6	0.482	NA	NA	NA	NA	NA	NA	**0.000**	**0.000**
Lee YH, 2012 [6S]	13	T vs. C	1.59 (1.486–1.69)	**<0.001**	F	RA	European	0.00	0.111	NA	NA	NA	NA	NA	NA	**0.000**	**0.000**
Lee YH, 2012 [6S]	5	CT +TT vs. CC	1.81 (1.28–2.56)	**<0.001**	R	RA	Non-European	61.3	0.647	0.011	0.146	0.988	1,000	0.855	1.000	0.984	1.000
Lee YH, 2012 [6S]	6	-	1.64 (1.44–1.87)	**<0.001**	F	RF+ vs. RF-	Overall mixed	0.00	0.24	0.000	0.095	**0.000**	**0.088**	**0.000**	**0.000**	**0.000**	**0.000**
Jiang Y, 2012 [7S]	34	TT vs. CC+TC	2.54 (2.17–2.98)	**<1.0 × 10^−15^**	F	RA	Overall mixed	0.00	-	NA	NA	NA	NA	NA	NA	**0.000**	**0.000**
Jiang Y, 2012 [7S]	10	T vs. C	1.67 (1.51–1.85)	**<1.0 × 10^−15^**	F	RF+	Overall mixed	0.00	-	NA	NA	NA	NA	NA	NA	**0.000**	**0.000**
Ramirez M, 2012 [8S]	2	T vs. C	1.90 (1.34–2.68)	**3.0 × 10^−4^**	F	RA	Overall mixed	-	-	0.004	0.089	0.983	1.000	0.741	1.000	0.969	1.000
Nong LM, 2011 [9S]	19	TT vs. CC	2.86 (2.29–3.57)	**<0.01**	-	RA	European	13.0	-	NA	NA	NA	NA	NA	NA	**0.000**	**0.000**
Nong LM, 2011 [9S]	6	TT vs. CC	4.49 (2.88–7.00)	**<0.01**	-	RF+	European	1.7	-	0.000	0.000	0.922	1.000	**0.049**	0.981	**0.058**	0.984
Nong LM, 2011 [9S]	6	TT vs. CC	2.86 (2.29–3.57)	**<0.01**	-	RF-	European	13.0	-	NA	NA	NA	NA	NA	NA	**0.000**	**0.000**
Nong LM, 2011 [9S]	19	T vs. C	1.54 (1.47–1.62)	**<0.01**	-	RA	Overall mixed	26,2	0.421	NA	NA	NA	NA	NA	NA	**0.000**	**0.000**
Nong LM, 2011 [9S]	-	T vs. C	1.70 (1.52–1.89)	**<0.01**	-	RF+	Overall mixed	0.00	>0.05	NA	NA	NA	NA	NA	NA	**0.000**	**0.000**
Nong LM, 2011 [9S]	-	T vs. C	1.37 (1.18–1.59)	**<0.01**	-	RF-	Overall mixed	45.3	>0.05	0.041	0.884	0.457	0.999	**0.037**	0.975	**0.601**	0.999
Totaro MC, 2011 [10S]	24	T vs. C	1.79 (1.60–2.01)	**<0.05**	R	RA	Overall mixed	79.42	0.46	NA	NA	NA	NA	NA	NA	**0.000**	**0.000**
Totaro MC, 2011 [10S]	23	T vs. C	1.80 (1.61–2.02)	**<0.05**	R	RA	Without Italian	79.42	-	NA	NA	NA	NA	NA	NA	**0.000**	**0.000**
Totaro MC, 2011 [10S]	14	T vs. C	2.01 (1.67–2.43)	**<0.05**	R	RA (miao)(Quality score >11)	Overall mixed	61.14	-	0.000	0.001	**0.011**	0.918	**0.000**	**0.000**	**0.000**	**0.001**
Plant P, 2010 [11S]	20	T vs. C	1.60 (1.53–1.67)	**2.30 × 10^−98^**	R	RA	Overall mixed	59.9	-	NA	NA	NA	NA	NA	NA	**0.000**	**0.000**
Plant P, 2010 [11S]	2	T vs. C	1.48 (1.33–1.66)	**6.25 × 10^−12^**	R	RA	Caucasian, European	>50	-	0.000	0.591	**0.000**	**0.112**	**0.000**	**0.000**	**0.000**	**0.004**
Curtin K, 2007 [12S]	2	CC vs. CT+TT	2.53 (1.32–4.84)	**<0.001**	-	RA	Overall mixed	NA	NA	0.012	0.057	0.998	1.000	0.989	1.000	0.997	1.000
Curtin K, 2007 [12S]	2	CC vs. CT+TT	2.13 (1.76–2.57)	**<0.001**	-	RF+	Overall mixed	NA	NA	0.000	0.000	**0.003**	0.731	**0.000**	**0.000**	**0.000**	**0.000**
Curtin K, 2007 [12S]	2	CC vs. CT+TT	1.90 (1.55–2.34)	**<0.001**	-	RA (CCP+)	Overall mixed	NA	NA	0.000	0.013	**0.168**	0.995	**0.000**	**0.106**	**0.001**	**0.542**
Curtin K, 2007 [12S]	2	CC vs. CT+TT	1.47 (1.15–1.88)	**0.001**	-	RA (CCP-)	Overall mixed	NA	NA	0.053	0.564	0.976	1.000	0.792	1.000	0.984	1.000
Lee YH, 2007 [13S]	12	TT vs. CT+CC	2.89 (2.19–3.82)	**<0.00001**	-	RA	Overall mixed	0.00	-	0.000	0.000	0.212	0.996	**0.000**	**0.042**	**0.000**	**0.011**

RA, rheumatoid arthritis; RF+, rheumatoid factor positive; RF−, rheumatoid factor negative; CCP+, cyclic citrullinated peptide positive; CCP−, cyclic citrullinated peptide negative; OR, odds ratio; CI, confidence interval; R, random; F, fixed; S, Supplementary; FPRP, false-positive report probability; BFDP, Bayesian false discovery probability. Each comparison of genetic associations was regarded as noteworthy when FPRP of <0.2 or BFDP of <0.8 or both are fulfilled and the values were bolded when the results are significant by FPRP or BFDP. NAs are expressed when information is not available by FPRP calculations.

**Table 2:** Meta-analysis results of associations between juvenile idiopathic arthritis (JIA) and the PTPN22 1858 C/T polymorphism from observational studies.

Author, Year	No. of Studies	Comparison	OR (95% CI)	*p*-Value	Model	Disease	Ethnicity	I^2^ (%)	Egger’s *p*-Value	Power OR 1.2	Power OR 1.5	FPRP Values at Prior Probability				BFDP 0.001	BFDP 0.000001
												OR 1.2		OR 1.5			
												0.001	0.000001	0.001	0.000001		
DI Y, 2015 [14S]	7	T vs. C	1.38 (1.04–1.83)	**0.025**	R	JIA (cases *n* < 500)	Overall mixed	72.4	0.619	0.166	0.719	0.993	1.000	0.972	1.000	0.997	1.000
DI Y, 2015 [14S]	4	T vs. C	1.55 (1.39–1.72)	**<0.001**	R	JIA (cases *n* ≥ 500)	Overall mixed	8.00	0.309	0.000	0.300	**0.000**	**0.000**	**0.000**	**0.000**	**0.000**	**0.000**
DI Y, 2015 [14S]	8	T vs. C	1.52 (1.32–1.76)	**<0.001**	R	JIA (population-based)	Overall mixed	55.5	0.979	0.001	0.430	**0.027**	0.965	**0.000**	**0.048**	**0.003**	**0.738**
DI Y, 2015 [14S]	3	T vs. C	1.36 (1.15–1.60)	**<0.001**	R	JIA (hospital-based)	Overall mixed	8.80	0.882	0.066	0.881	0.761	1.000	**0.191**	0.996	0.878	1.000
DI Y, 2015 [14S]	4	T vs. C	1.52 (1.32–1.78)	**<0.001**	F	JIA	American	45.7	0.585	0.002	0.435	**0.108**	0.992	**0.000**	0.317	**0.022**	0.957
DI Y, 2015 [14S]	6	T vs. C	1.36 (1.01–1.83)	**0.041**	R	JIA	Augean	74.4	0.555	0.204	0.741	0.995	1.000	0.983	1.000	0.998	1.000
DI Y, 2015 [14S]	11	T vs. C	1.42 (1.20–1.68)	**<0.001**	R	JIA	Overall mixed	61.6	0.303	0.025	0.739	0.636	0.999	**0.056**	0.983	**0.676**	1.000
DI Y, 2015 [14S]	9	T vs. C	1.48 (1.36–1.62)	**<0.001**	F	JIA (Adjusted)	Overall mixed	8.8	0.268	NA	NA	NA	NA	NA	NA	**0.000**	**0.000**
Kaalla M, 2013 [15S]	8	T vs. C	1.44 (1.31, 1.60)	**<0.0001**	F	RA	Overall mixed	-	-	0.000	0.776	**0.000**	**0.033**	**0.000**	**0.000**	**0.000**	**0.002**
Kaalla M, 2013 [15S]		T vs. C	2.05 (1.37, 3.77)	**<0.0005**	-	RF+	Overall mixed	-	-	0.042	0.157	0.998	1.000	0.993	1.000	0.998	1.000
Kaalla M, 2013 [15S]		T vs. C	1.56 (1.21, 2.02)	**0.0007**	-	RF−	Overall mixed	-	-	0.023	0.383	0.970	1.000	0.660	0.999	0.970	1.000
Kaalla M, 2013 [15S]		T vs. C	1.45 (1.18, 1.79)	**<0.0005**	-	RA (Oligoarticular)	Overall mixed	-	-	0.039	0.624	0.933	1.000	0.466	0.999	0.951	1.000
Zheng J, 2012 [5S]	7	T vs. C	1.54 (1.40–1.70)	**1.0 × 10^−16^**	-	JIA	Overall mixed	-	-	NA	NA	NA	NA	NA	NA	**0.000**	**0.000**
Lee YH, 2012[16S]	7	T vs. C	1.311 (1.205–1.427)	**<1.8 × 10^−8^**	-	JIA	European	32.2	0.353	0.020	0.999	**0.000**	**0.019**	**0.000**	**0.000**	**0.000**	**0.033**
Lee YH, 2007 [13S]	2	TT vs. CC	1.89 (1.09–3.29)	**0.02**	-	JIA	Overall mixed	0.00	-	0.054	0.207	0.998	1.000	0.992	1.000	0.998	1.000

JIA, juvenile idiopathic arthritis; OR, odds ratio; R, random; F, fixed; S, supplementary; FPRP, false-positive report probability; BFDP, Bayesian false discovery probability. Each comparison of genetic associations was regarded as noteworthy when FPRP of <0.2 or BFDP of <0.8 or both are fulfilled and the values were bolded when the results are significant by FPRP or BFDP. NAs are expressed when information is not available by FPRP calculations.

**Table 3:** Meta-analysis results of associations between systemic lupus erythematosus (SLE) and the PTPN22 1858 C/T polymorphism from observational studies.

Author, Year	No. of Studies	Comparison	OR (95% CI)	*p*-Value	Model	Disease	Ethnicity	I^2^ (%)	Egger’s *p*-Value	Power OR 1.2	Power OR 1.5	FPRP Values at Prior Probability				BFDP 0.001	BFDP 0.000001
												OR 1.2		OR 1.5			
												0.001	0.000001	0.001	0.000001		
Hu LY, 2017 [17S]	17	TT+CT vs. CC	1.53 (1.346–1.742)	**9.17 × 10^−11^**	R	SLE	Overall mixed	44.2	0.05	0.000	0.378	**0.001**	0.481	**0.000**	**0.000**	**0.000**	**0.019**
Hu LY, 2017 [17S]	4	T vs. C	2.56 (1.796–3.665)	**2.219 × 10^−7^**	F	SLE	American	0.00	>0.05	0.000	0.002	0.938	1.000	**0.122**	0.993	**0.438**	0.999
Hu LY, 2017 [17S]	11	T vs. C	1.39 (1.261–1.552)	**2.153 × 10^−10^**	F	SLE	European	32.8	0.938	0.002	0.906	**0.000**	**0.108**	**0.000**	**0.000**	**0.000**	**0.025**
Hu LY, 2017 [17S]	2	TT+CT vs. CC	5.08 (2.053–12.569)	**4.315 × 10^−4^**	F	SLE	African	0.00	>0.05	0.001	0.004	0.998	1.000	0.991	1.000	0.997	1.000
de Lima SC, 2017 [18S]	19	T vs. C	1.54 (1.38–1.72)	**0.000**	F	SLE	Overall mixed	-	-	0.000	0.320	**0.000**	**0.004**	**0.000**	**0.000**	**0.000**	**0.000**
de Lima SC, 2017 [18S]	NA	T vs. C	1.47 (1.29–1.66)	**0.0000**	F	SLE	Caucasian	13.96	-	0.001	0.628	**0.001**	0.495	**0.000**	**0.001**	**0.000**	**0.067**
de Lima SC, 2017 [18S]	NA	T vs. C	2.41 (1.68–3.44)	**0.0000**	F	SLE	Latin	0.000	-	0.000	0.005	0.954	1.000	0.219	0.996	**0.667**	1.000
Shi L, 2013 [19S]	4	T vs. C	2.33 (1.78–3.04)	**<0.00001**	F	SLE	Overall mixed	0.00	<0.05	0.000	0.001	0.475	0.999	**0.001**	0.438	**0.002**	**0.698**
Shi L, 2013[19S]	4	TC vs. CC+TT	2.33 (1.78–3.04)	**< 0.00001**	F	SLE	Overall mixed	0.00	-	0.000	0.001	0.475	0.999	**0.001**	0.438	**0.002**	**0.698**
Lea WW, 2011 [20S]	11	T vs. C	1.56 (1.34–1.822)	**2.0× 10^−8^**	R	SLE	Overall mixed	49.3	0.405	0.000	0.310	**0.041**	0.977	**0.000**	**0.060**	**0.003**	**0.750**
Zheng J, 2012 [5S]	14	T vs. C	1.46 (1.31–1.62	**7.33 × 10^−13^**	-	SLE	Overall mixed	-	-	0.000	0.695	**0.000**	**0.009**	**0.000**	**0.000**	**0.000**	**0.000**
Ramirez M, 2012 [8S]	2	T vs. C	2.65 (0.74–4.05)	**1.0 × 10^−5^**	F	SLE	Overall mixed	NA	-	0.000	0.004	0.982	1.000	0.610	0.999	0.916	1.000
Lea WW, 2011 [20S]	7	T vs. C	1.49 (1.28–1.735)	**2.8 × 10^−8^**	R	SLE	European	46.5	0.908	0.003	0.534	**0.096**	0.991	**0.001**	0.346	**0.027**	0.965
Lea WW, 2011 [20S]	3	T vs. C	2.35 (1.644–3.373)	**2.9 × 10^−6^**	F	SLE	Hispanic	0.00	0.639	0.000	0.007	0.962	1.000	0.300	0.998	**0.769**	1.000
Lee YH, 2007 [13S]	5	CT vs. CC	1.41 (1.22–1.63)	**<0.00001**	-	SLE	Overall mixed	0.00	-	0.015	0.799	**0.189**	0.996	**0.004**	0.810	**0.172**	0.995

SLE, systemic lupus erythematosus; OR, odds ratio; R, random; F, fixed; S, supplementary; FPRP, false-positive report probability; BFDP, Bayesian false discovery probability. Each comparison of genetic associations was regarded as noteworthy when FPRP of <0.2 or BFDP of <0.8 or both are fulfilled and the values were bolded when the results are significant by FPRP or BFDP.

**Table 4:** Meta-analysis results of associations between vasculitides and the PTPN22 1858 C/T polymorphism from observational studies.

Author, Year	No. of Studies	Comparison	OR (95% CI)	*p*-Value	Model	Disease	Ethnicity	I^2^ (%)	Egger’s *p*-Value	Power OR 1.2	Power OR 1.5	FPRP Values at Prior Probability				BFDP 0.001	BFDP 0.000001
												OR 1.2		OR 1.5			
												0.001	0.000001	0.001	0.000001		
**ANCA-associated vasculitis**																	
Rahmattulla, 2015 [21S]	4	CT +TT vs. CC	1.39 (1.24–1.56)	**<0.001**	R	ANCA-associated vasculitis	Overall mixed	0.00	-	0.006	0.902	**0.004**	0.780	**0.000**	**0.024**	**0.002**	**0.654**
Cao Y, 2015 [22S]	4	A vs. G	1.44 (1.26–1.64)	**2.27 × 10^−5^**	F	ANCA	White population	0.00	>0.05	0.003	0.731	**0.013**	0.929	**0.000**	**0.051**	**0.004**	**0.788**
Cao Y, 2015 [22S]	3	A vs. G	1.72 (1.35–2.20)	**1.25 × 10^−7^**	F	GPA	White population	0.00	>0.05	0.002	0.138	0.883	1.000	**0.102**	0.991	**0.679**	1.000
Cao Y, 2015 [22S]	2	A vs. G	1.53 (1.08–2.15)	**0.02**	F	MPA	White population	0.00	>0.05	0.081	0.455	0.994	1.000	0.969	1.000	0.996	1.000
Cao Y, 2015 [22S]	2	A vs. G	1.74 (1.25–2.43)	**0.001**	F	ANCA(miao)(with proteinase 3)	White population	0.00	>0.05	0.015	0.192	0.987	1.000	0.857	1.000	0.985	1.000
Cao Y, 2015 [22S]	2	A vs. G	1.94 (0.64–5.85)	**0.24**	R	ANCA(miao)(myeloperoxidase)	White population	77	>0.05	0.197	0.324	0.999	1.000	0.999	1.000	0.999	1.000
Lee YH, 2012 [23S]	2	T vs. C	2.04 (1.53–2.719)	**1.02 × 10^−6^**	F	ANCA+WG	Caucasian	0	NA	0.000	0.017	0.882	1.000	**0.056**	0.983	**0.375**	0.998
Lee YH, 2012 [23S]	2	T vs. C	3.43 (1.18–10.36)	**0.029**	F	ANCA+ vs. ANCA	Overall mixed	0	NA	0.031	0.071	0.999	1.000	0.998	1.000	0.999	1.000
Lee YH, 2012 [23S]	3	T vs. C	1.41 (1.23–1.63	**1.59 × 10^−6^**	F	AAV	Caucasian	0.00	0.481	0.011	0.791	**0.119**	0.993	**0.002**	0.656	**0.091**	0.990
Lee YH, 2012 [23S]	2	T vs. C	1.83 (1.37–2.43)	**3.09 × 10^−5^**	F	WG	Caucasian	0	NA	0.002	0.086	0.945	1.000	0.271	0.997	0.841	1.000
Zheng J, 2012 [5S]	2	T vs. C	1.45 (1.24–1.69)	**3.07 × 10^−6^**	-	ANCA-associated(miao)vasculitis	Overall mixed	-	-	0.008	0.668	0.204	0.996	**0.003**	0.748	**0.125**	0.993
**Giant cell arteritis**																	
Lester S, 2016 [24S]	7	T vs. C	1.33 (1.16–1.52)	**3.0 × 10^−5^**	R	GCA	Overall mixed	4.72	-	0.066	0.961	0.302	0.998	**0.029**	0.967	**0.541**	0.999
Lester S, 2016 [24S]	5	T vs. C	1.21 (1.03–1.43)	**<0.05**	R	GCA	Northern European	-	-	0.461	0.994	0.982	1.000	0.962	1.000	0.997	1.000
Lester S, 2016 [24S]	2	T vs. C	1.56 (1.28–1.91)	**<0.05**	R	GCA	Southern European	-	-	0.006	0.352	0.750	1.000	**0.045**	0.979	**0.572**	0.999

ANCA, anti-neutrophil cytoplasmic antibody; AAV, ANCA-associated vasculitis; WG, Wegener’s granulomatosis; GCA, giant cell arteritis; GPA, granulomatosis with polyangiitis; MPA, microscopic polyangiitis; OR, odds ratio; R, random; F, fixed; S, supplementary; FPRP, false-positive report probability; BFDP, Bayesian false discovery probability. Each comparison of genetic associations was regarded as noteworthy when FPRP of <0.2 or BFDP of <0.8 or both are fulfilled and the values were bolded when the results are significant by FPRP or BFDP.

**Table 5:** Meta-analysis results of associations between other rheumatic diseases and the PTPN22 1858 C/T polymorphism from observational studies.

Author, Year	No. of Studies	Comparison	OR (95% CI)	*p*-Value	Model	Disease	Ethnicity	I^2^ (%)	Egger’s *p*-Value	Power OR 1.2	Power OR 1.5	FPRP Values at Prior Probability				BFDP 0.001	BFDP 0.000001
												OR 1.2		OR 1.5			
												0.001	0.000001	0.001	0.000001		
**Systemic sclerosis**																	
Zheng J, 2012 [5S]	11	T vs. C	1.16 (1.02–1.31)	**0.015**	-	SSc	Overall mixed	-	-	0.708	1.000	0.959	1.000	0.944	1.000	0.996	1.000
Zheng J, 2012 [5S]	11	T vs. C	1.25 (1.03–1.54)	**0.037**	-	ATA+SSc	Overall mixed	-	-	0.351	0.957	0.990	1.000	0.974	1.000	0.997	1.000
Zheng J, 2012 [5S]	11	T vs. C	1.16 (1.00–1.33)	**0.019**	-	ATA-SSc	Overall mixed	-	-	0.686	1.000	0.980	1.000	0.971	1.000	0.998	1.000
Diaz-Gallo LM, 2011 [25S]	9	T vs. C	1.15 (1.03–1.28)	**0.03**	F	SSc	Overall mixed	33.8	-	0.782	1.000	0.931	1.000	0.913	1.000	0.995	1.000
Diaz-Gallo LM, 2011 [25S]	9	T vs. C	1.22 (1.05–1.42)	**0.02**	F	SSc(ACA+)	Overall mixed	0.0	-	0.416	0.996	0.961	1.000	0.911	1.000	0.994	1.000
Dieudé P, 2007 [26S]	3	CT+TT vs. CC	1.08 (1.02–1.15)	**8.39 × 10^−3^**	NA	SSc	Caucasian	NA	-	0.999	1.000	0.942	1.000	0.942	1.000	0.997	1.000
Dieudé P, 2007 [26S]	3	CT+TT vs. CC	1.09 (1.04–1.16)	**3.11 × 10^−3^**	NA	SSc	Overall mixed	NA	-	0.999	1.000	0.869	1.000	0.869	1.000	0.994	1.000
**Psoriasis**																	
Chen YF, 2012 [27S]	10	T vs. C	1.15 (1.00–1.33)	**<0.05**	R	Ps	Overall mixed	27.8	<0.05	0.717	1.000	0.988	1.000	0.983	1.000	0.998	1.000
Chen YF, 2012 [27S]	5	T vs. C	1.23 (1.00–1.52)	**<0.05**	R	Ps	Overall mixed	36.4	-	0.410	0.967	0.993	1.000	0.983	1.000	0.998	1.000
Zheng J, 2012 [5S]	4	T vs. C	1.22 (1.02–1.44)	**0.023**	-	Ps	Overall mixed	-	-	0.423	0.993	0.978	1.000	0.950	1.000	0.996	1.000
**Ankylosing spondylitis**																	
Meng W, 2017 [28S]	3	TT vs. CC	1.67 (0.39, 7.06)	**>0.05**	F	AS	Overall mixed	0.00	-	0.327	0.442	0.999	1.000	0.999	1.000	0.999	1.000
**Sjögren’s syndrome**																	
Zheng J, 2012 [5S]	2	T vs. C	1.40 (0.91–2.14)	**0.01**	-	SS	Overall mixed	-	-	0.238	0.625	0.998	1.000	0.995	1.000	0.999	1.000

ATA, anti-topoisomerase I antibodies; ACA, anticentromere antibodies; SSc, systemic sclerosis; AS, ankylosing spondylitis; SS, Sjögren’s syndrome; Ps, psoriasis; OR, odds ratio; R, random; F, fixed; S, supplementary; FPRP, false-positive report probability; BFDP, Bayesian false discovery probability. Each comparison of genetic associations was regarded as noteworthy when FPRP of <0.2 or BFDP of <0.8 or both are fulfilled and the values were bolded when the results are significant by FPRP or BFDP.

**Table 6:** Meta-analysis results of associations between non-rheumatic autoimmune or autoimmunity-related diseases and the PTPN22 1858 C/T polymorphism from observational studies.

Author, Year	No. of Studies	Comparison	OR (95% CI)	*p*-Value	Model	Disease	Ethnicity	I^2^ (%)	Egger’s *p*-Value	Power OR 1.2	Power OR 1.5	FPRP Values at Prior Probability				BFDP 0.001	BFDP 0.000001
												OR 1.2		OR 1.5			
												0.001	0.000001	0.001	0.000001		
**Vitiligo**																	
Agarwal S, 2017 [29S]	7	T vs. C	1.50 (1.32–1.71)	**<0.001**	F	Vitiligo	Overall mixed	35.0	-	0.000	0.500	**0.003**	0.757	**0.000**	**0.003**	**0.000**	**0.160**
Agarwal S, 2017 [29S]	3	T vs. C	1.53 (1.34–1.75)	**<0.00001**	F	Vitiligo	European	32.0	-	0.000	0.386	**0.003**	0.736	**0.000**	**0.001**	**0.000**	**0.085**
Song GG, 2013 [30S]	5	T vs. C	1.507 (1.320–1.720)	**<1.0 × 10^−8^**	F	Vitiligo	Overall mixed	<50	>0.05	0.000	0.472	**0.003**	0.766	**0.000**	**0.003**	**0.000**	**0.152**
Song GG, 2013 [30S]	4	T vs. C	1.530 (1.339–1.748)	**<1.0 × 10^−8^**	F	Vitiligo	European	0<50	>0.05	0.000	0.385	**0.002**	0.691	**0.000**	**0.001**	**0.000**	**0.064**
Zheng J, 2012 [5S]	2	T vs. C	1.98 (1.35–2.88)	**3.70 × 10^−4^**	-	GV	Overall mixed	-	-	0.004	0.073	0.988	1.000	0.828	1.000	0.979	1.000
**Crohn’s disease**																	
Hedjoudje A, 2017 [31S]	13	T vs. C	1.28 (1.17–1.4)	**8.48 × 10^−8^**	F	CD	Overall mixed	37,54	0.48	0.079	1.000	**0.001**	0.458	**0.000**	**0.063**	**0.004**	0.810
Li X, 2017 [32S]		-	0.61 (0.44–0.84)	**0.002**	R	CD	Overall mixed	78	-	0.028	0.293	0.989	1.000	0.893	1.000	0.990	1.000
Zheng J, 2012 [5S]	11	T vs. C	0.84 (0.76–0.94)	**1.89 × 10^−3^**	-	CD	Overall mixed	-	-	0.555	1.000	0.811	1.000	0.704	1.000	0.981	1.000
Diaz-Gallo, 2011 [33S]	12	T vs. C	0.81 (0.75 0.89)	**7.4 × 10^−6^**	F	CD	European	NA	-	0.277	1.000	**0.040**	0.977	**0.011**	0.921	**0.325**	0.998
Latiano, 2007 [34S]	4	TT+ CT vs. CC	0.77 (0.61–0.97)	**0.028**	F	CD	Overall mixed	<52	-	0.251	0.889	0.991	1.000	0.968	1.000	0.997	1.000
**Inflammatory bowel disease**																	
Li X, 2017 [32S]	10	-	0.71 (0.56–0.90)	**0.005**	R	IBD	Overall	81	0.187	0.093	0.699	0.980	1.000	0.869	1.000	0.990	1.000
**Myasthenia gravis**																	
Xiong X, 2015 [35S]	7	-	1.57 (1.34–1.82)	**<0.00001**	R	MG	Overall mixed	31	NA	0.000	0.273	**0.012**	0.923	**0.000**	**0.008**	**0.000**	**0.286**
Provenzano C, 2012 [36S]	4	T vs. C	1.56 (1.24–1.95)	**<1.0 × 10^−4^**	R	MG	Overall mixed	14	-	0.011	0.365	0.898	1.000	0.204	0.996	0.856	1.000
Provenzano C, 2012 [36S]	4	T vs. C	1.64 (1.40–1.93)	**<1.00 × 10^−5^**	R	MG (AChR+)	Overall mixed	14	-	0.000	0.141	**0.030**	0.968	**0.000**	**0.018**	**0.001**	**0.386**
Provenzano C, 2012 [36S]	4	T vs. C	1.82 (1.44–2.28)	**<1.00 × 10^−5^**	R	MG (thymoma-)	Overall mixed	14	-	0.000	0.046	0.566	0.999	**0.004**	0.804	**0.061**	0.985
Zheng J, 2012 [5S]	5	T vs. C	1.53 (1.31–1.80)	**1.09 × 10^−7^**	-	MG	Overall mixed	-	-	0.002	0.406	**0.147**	0.994	**0.001**	0.418	**0.031**	0.970
**Behçet’s disease**																	
Lee YH, 2012 [23S]	3	T vs. C	0.388 (0.916–0.770)	**0.007**	F	BD	Caucasian	55.9	0.104	0.014	0.061	0.998	1.000	0.991	1.000	0.998	1.000
Lee YH, 2012 [23S]	1	T vs. C	0.37 (0.179–0.765)	**0.007**	NA	BD	European	NA	NA	0.014	0.056	0.998	1.000	0.992	1.000	0.998	1.000
**Autoimmune thyroid disease**																	
Luo L, 2012 [37S]	11	TT+TC vs. CC	1.41 (1.12,1.78)	**0.07**	R	AITD	Overall mixed	-	>0.05	0.087	0.699	0.978	1.000	0.846	1.000	0.989	1.000
Luo L, 2012 [37S]	7	TT+TC vs. CC	1.41 (1.09,1.83)	**0.03**	R	AITD	Caucasian	-	>0.05	0.113	0.679	0.989	1.000	0.935	1.000	0.994	1.000
Luo L, 2012 [37S]	4	TT+TC vs. CC	1.01 (0.51,2.00)	**0.01**	R	AITD	Others	-	>0.05	0.690	0.872	0.999	1.000	0.999	1.000	0.999	1.000
**Graves’ disease**																	
Luo L, 2012 [37S]	8	TC vs. CC	1.46 (1.12,1.89)	**0.07**	R	GD	Overall mixed	-	>0.05	0.068	0.581	0.983	1.000	0.875	1.000	0.990	1.000
Zheng J, 2012 [5S]	3	T vs. C	1.59 (1.37–1.85)	**1.01 × 10^−9^**	-	GD	Overall mixed	-	-	0.000	0.225	**0.014**	0.935	**0.000**	**0.009**	**0.000**	**0.281**
Lee YH, 2007 [13S]	3	CT vs. CC	1.66 (1.35–2.04)	**<0.00001**	-	GD	Overall mixed	28.1	-	0.001	0.168	0.586	0.999	**0.009**	0.896	**0.178**	0.995
**Addison’s disease**																	
Zheng J, 2012 [5S]	6	T vs. C	1.43 (1.21–1.68)	**2.36 × 10^−5^**	-	AD	Overall mixed	-	-	0.016	0.720	0.451	0.999	**0.018**	0.950	**0.432**	0.999
Skinningsrud B, 2008 [38S]	4	T vs. C	1.36 (1.11–1.66)	**0.003**	-	AD	European	<50	-	0.109	0.832	0.958	1.000	0.750	1.000	0.983	1.000
Roycroft M, 2009 [39S]	5	T vs. C	1.44 (1.21–1.72)	**5.6 × 10^−5^**	F	AD	Caucasian, European	0.00	-	0.022	0.674	0.722	1.000	**0.079**	0.988	**0.738**	1.000
**Endometriosis**																	
Pabalan N, 2017 [40S]	10	Co-dominant	3.14 (1.93–5.10)	**<0.001**	-	Endometriosis	Overall mixed	86.0	-	0.000	0.001	0.987	1.000	0.727	1.000	0.941	1.000
Pabalan N, 2017 [40S]	9	Co-dominant	3.08 (1.84–5.14)	**<0.001**	-	Endometriosis (HWE only)	Overall mixed	88.0	-	0.000	0.003	0.991	1.000	0.850	1.000	0.973	1.000
Pabalan N, 2017 [40S]	8	Co-dominant	3.86 (2.40–6.21)	**<0.001**	-	Endometriosis	Italian	78.0	-	0.000	0.000	0.972	1.000	0.346	0.998	**0.658**	0.999
**Alopecia areata**																	
Zheng J, 2012 [5S]	2	T vs. C	1.38 (1.11–1.72)	**0.003**	-	AA	Overall mixed	-	-	0.107	0.771	0.975	1.000	0.843	1.000	0.989	1.000

GV, generalized vitiligo; BD, Behçet’s disease; IBD, inflammatory bowel disease; AD, Addison’s disease; MG, myasthenia gravis; GD, Graves’ disease; Crohn’s disease; AITD, autoimmune thyroid disease; AA, alopecia areata; OR, odds ratio; R, random; F, fixed; S, supplementary; FPRP, false-positive report probability; BFDP, Bayesian false discovery probability. Each comparison of genetic associations was regarded as noteworthy when FPRP of <0.2 or BFDP of <0.8 or both are fulfilled and the values were bolded when the results are significant by FPRP or BFDP.

**Table 7:** Meta-analysis results of associations between type 1 diabetes mellitus (T1DM) and the PTPN22 1858 C/T polymorphism from observational studies.

Author, Year	No. of Studies	Comparison	OR (95% CI)	*p*-Value	Model	Disease	Ethnicity	I^2^ (%)	Egger’s *p*-Value	Power OR 1.2	Power OR 1.5	FPRP Values at Prior Probability				BFDP 0.001	BFDP 0.000001
												OR 1.2		OR 1.5			
												0.001	0.000001	0.001	0.000001		
Ramu D, 2018 [41S]	16	TT vs. CC	2.67 (1.92–3.70)	**<0.0001**	F	T1DM (LADA)	Overall mixed	24.8	-	0.000	0.000	0.824	1.000	**0.013**	0.932	**0.040**	0.976
Dong F, 2014 [42S]	5	T vs. C	1.52 (1.29–1.79)	**<0.001**	F	T1DM (LADA)	Overall mixed	12.14	0.54	0.002	0.437	**0.184**	0.996	**0.001**	0.543	**0.050**	0.981
Xuan C, 2013 [43S]	28	CT+TT vs. CC	1.957 (1.817–2.108)	**2.94 × 10^−70^**	F	T1DM	Overall mixed	36.7	0.544	NA	NA	NA	NA	NA	NA	**0.000**	**0.000**
Xuan C, 2013 [43S]	27	CT+TT vs. CC	1.962 (1.821–2.113)	**2.46 × 10^−70^**	R	T1DM	Caucasian	37.5	0.320	NA	NA	NA	NA	NA	NA	**0.000**	**0.000**
Xuan C, 2013 [43S]	7	CT+TT vs. CC	1.96 (1.806–2.127)	**1.85 × 10^−58^**	F	T1DM(Male)	Caucasian	0.00	0.548	NA	NA	NA	NA	NA	NA	**0.000**	**0.000**
Xuan C, 2013 [43S]	7	TT vs. CC	3.537 (2.704–4.625)	**2.71 × 10^−20^**	F	T1DM(Female)	Caucasian	31.8	0.764	NA	NA	NA	NA	NA	NA	**0.000**	**0.000**
Wang X F, 2013 [44S]	34	Recessive	2.78 (2.25–3.44)	**<1.0 × 10^−5^**	R	T1DM	Overall mixed	NA	NA	NA	NA	NA	NA	NA	NA	**0.000**	**0.000**
Wang XF, 2013 [44S]	23	Recessive	3.42 (2.55–4.59)	**<1.0 × 10^−5^**	R	T1DM (<500)	Overall mixed	NA	NA	0.000	0.000	**0.106**	0.992	**0.000**	**0.009**	**0.000**	**0.000**
Wang XF, 2013 [44S]	8	Recessive	2.27 (1.71–3.01)	**<1.0 × 10^−5^**	R	T1DM (500~1000)	Overall mixed	NA	NA	0.000	0.002	0.722	1.000	**0.006**	0.861	**0.031**	0.969
Wang XF, 2013 [44S]	3	Recessive	2.26 (1.57–3.25)	**<1.0 × 10^−5^**	R	T1DM (>1000)	Overall mixed	NA	NA	0.000	0.014	0.972	1.000	0.446	0.999	0.875	1.000
Wang XF, 2013 [44S]	8	allelic	1.80 (1.36–6.55)	**<1.0 × 10^−5^**	R	T1DM (Male)	Overall mixed	NA	NA	0.269	0.391	0.999	1.000	0.999	1.000	0.999	1.000
Wang XF, 2013 [44S]	8	allic	8.26 (3.05–22.38)	**<1.0 × 10^−5^**	R	T1DM (Female)	Overall mixed	NA	NA	0.000	0.000	0.998	1.000	0.988	1.000	0.997	1.000
Wang XF, 2013 [44S]	20	Recessive	2.57 (2.00–3.32)	**<1.0 × 10^−5^**	R	T1DM (Early)	Overall mixed	NA	NA	0.000	0.000	**0.153**	0.994	**0.000**	**0.026**	**0.000**	**0.015**
Wang XF, 2013 [28S]	8	Recessive	5.86 (3.40–10.12)	**<1.0 × 10^−5^**	R	T1DM (Late)	Overall mixed	NA	NA	0.000	0.000	0.972	1.000	0.307	0.998	**0.495**	0.999
Tang S, 2012 [45S]	24	CC vs. CT	0.532 (0.467–0.595)	**<0.001**	R	T1DM	Overall mixed	38.47	0.695	NA	NA	NA	NA	NA	NA	**0.000**	**0.000**
Tang S, 2012 [45S]	18	CC vs. CT	0.532 (0.467–0.606)	**<0.05**	-	T1DM	Europe	NA	>0.05	NA	NA	NA	NA	NA	NA	**0.000**	**0.000**
Tang S, 2012 [45S]	4	CC vs. CT	0.526 (0.434–0.636)	**<0.05**	-	T1DM	America	NA	>0.05	0.000	0.007	**0.032**	0.970	**0.000**	**0.005**	**0.000**	**0.034**
Peng H, 2012 [46S]	24	TT+TC vs. CC	1.988 (1.832–2.157)	**<0.001**	R	T1DM	Overall mixed	34.8	0.560	NA	NA	NA	NA	NA	NA	**0.000**	**0.000**
Peng H, 2012 [46S]	19	TT+TC vs. CC	1.976 (1.801–2.169)	**<0.001**	R	T1DM	European	40.2	0.355	NA	NA	NA	NA	NA	NA	**0.000**	**0.000**
Peng H, 2012 [46S]	5	TT+TC vs. CC	2.017 (1.727,2.355)	**<0.001**	F	T1DM	American	14.0	0.486	NA	NA	NA	NA	NA	NA	**0.000**	**0.000**
Zheng J, 2012 [5S]	23	T vs. C	1.84 (1.72–1.96)	**<1.0 × 10^−16^**	-	T1DM	Overall mixed	-	-	NA	NA	NA	NA	NA	NA	**0.000**	**0.000**
Lee YH, 2007 [13S]	6	TT*vs*.CC	3.56 (2.39–5.31)	**<0.00001**	-	T1DM	Overall mixed	0.00	-	0.000	0.000	0.908	1.000	**0.041**	0.977	**0.072**	0.987

T1DM, type 1 diabetes mellitus; LADA, latent autoimmune diabetes in adults; OR, odds ratio; R, random; F, fixed; S, supplementary; FPRP, false-positive report probability; BFDP, Bayesian false discovery probability. Each comparison of genetic associations was regarded as noteworthy when FPRP of <0.2 or BFDP of <0.8 or both are fulfilled and the values were bolded when the results are significant by FPRP or BFDP. NAs are expressed when information is not available by FPRP calculations.

**Table 8:** Meta-analysis results of gene variants from genome-wide association studies showing significant *p*-value (<5 × 10^−8^).

Author, Year	No. of Studies	Comparison	OR (95% CI)	*p*-Value	Model	Disease	Ethnicity	I^2^ (%)	Power OR 1.2	Power OR 1.5	FPRP Values at Prior Probability				BFDP 0.001	BFDP 0.000001
											OR 1.2		OR 1.5			
											0.001	0.000001	0.001	0.000001		
Bowes J, 2014 [47S]	4	T vs. C	1.32 (1.21–1.45)	**1.49** **× 10^−9^**	-	PsA	Caucasian	0.023	0.996	0.000	0.228	**0.000**	**0.007**	**0.023**	**0.001**	**0.349**
Gregersen PK, 2012 [48S]	3	T vs. C	1.71 (1.44–2.02)	**3.72** **× 10^−10^**	-	MG	North European	0.000	0.062	0.018	0.947	**0.000**	**0.004**	**0.000**	**0.000**	**0.095**
Thompson SD, 2010 [49S]	2	additive	1.64 (1.44–1.87)	**1.90** **× 10^−13^**	-	JIA	Initial + Replication	0.000	0.091	0.000	**0.088**	**0.000**	**0.000**	**0.000**	**0.000**	**0.000**
Coenen MJ, 2009 [50S]	2	T vs. C	1.67 (1.52–1.84)	**2.0** **× 10^−27^**	R	RA	Caucasian European(miao)(Dutch+ UK)	NA	NA	NA	NA	NA	NA	NA	**0.000**	**0.000**

RA, rheumatoid arthritis; PsA, psoriatic arthritis; MG, myasthenia gravis; JIA, juvenile idiopathic arthritis; OR, odds ratio; R, random; S, supplementary; FPRP, false-positive report probability; BFDP, Bayesian false discovery probability. Each comparison of genetic associations was regarded as noteworthy when FPRP of <0.2 or BFDP of <0.8 or both are fulfilled and the values were bolded when the results are significant by FPRP or BFDP. NAs are expressed when information is not available by FPRP calculations.

**Table 9:** Meta-analysis results of the PTPN22 1858 C/T polymorphism from genome wide association studies showing significant *p*-value (5 × 10^−8^ < *p*< 0.05).

Author, Year	NO. of studies	Comparison	OR (95% CI)	*p*-Value	Model	Disease	Ethnicity	I^2^ (%)	Power OR 1.2	Power OR 1.5	FPRP Values at Prior Probability				BFDP 0.001	BFDP 0.000001
											OR 1.2		OR 1.5			
											0.001	0.000001	0.001	0.000001		
Merkel PA, 2017 [51S]	3	T vs. C	1.36 (1.21–1.53)	**1.86** **× 10^−7^**	-	ANCA	Overall European ancestry	0.019	0.948	0.016	0.943	**0.000**	0.247	**0.019**	**0.020**	0.953
Törn C, 2015 [52S]	3	T vs. C	2.42 (1.70–3.44)	**1.01** **× 10^−6^**	-	T1DM vs. Ab-	Caucasian children	0.000	0.004	0.948	1,000	**0.180**	0.995	**0.000**	**0.601**	0.999
Serrano A, 2013	4	T vs. C	1.51 (1.28–1.79)	**2.0** **× 10^−6^**	F	GCA	Caucasian	0.004	0.469	0.336	0.998	**0.004**	0.814	**0.004**	**0.151**	0.994
Thompson SD, 2010 [53S]	3	Recessive	1.63 (1.35–1.97)	**3.12** **× 10^−7^**	-	JIA	Germany +Texas +Utah	0.001	0.195	0.360	0.998	**0.002**	0.689	**0.001**	**0.062**	0.985

GCA, giant cell arteritis; PsA, psoriatic arthritis; JIA, juvenile idiopathic arthritis; RA, rheumatoid arthritis; ANCA, antineutrophil cytoplasmic antibody; GPA, granulomatosis with polyangiitis; MPA, microscopic polyangiitis; PR3, proteinase 3; cANCA, cytoplasmic ANCA; MPO, myeloperoxidase; pANCA, perinuclear ANCA; MG, myasthenia gravis; OR, odds ratio; R, random; FPRP, false-positive report probability; BFDP, Bayesian false discovery probability. Each comparison of genetic associations was regarded as noteworthy when FPRP of <0.2 or BFDP of <0.8 or both are fulfilled and the values were bolded when the results are significant by FPRP or BFDP.

## Supplementary Materials

The following are available online at https://www.mdpi.com/2077-0383/8/3/347/s1, Table S1: Genotypic and allelic comparisons from observational studies with non-significant *p*-value (>0.05), Table S2: Genotypic and allelic comparisons from GWAS studies not showing 95% CI, Table S3: PRISMA Checklist, Supplementary references.
