# Supplementary material for: Association of PTPN22 1858C/T Polymorphism with Autoimmune Diseases: A Systematic Review and Bayesian Approach
Source: J Clin Med. 2019 Mar 12;8(3):347. doi: 10.3390/jcm8030347 (PMC6462981; doi:10.3390/jcm8030347)
Supplement: Supplementary file 1 [file jcm-08-00347-s001.pdf]

Supplements

Association of PTPN22 1858C/T polymorphism with autoimmune diseases:

A systematic review and Bayesian approach

Table S1. Genotypic and allelic comparisons from observational studies with non-significant *p*-value (>0.05).

Author, Year	NO. of studies	Gene/Variant	Comparison	OR (95% CI)	*p*-value	Model	Disease	Ethnicity	No. of Cases/Controls	I² (%)	I² (P)	Egger's *p*-value
Cao Y, 2015	2	PTPN22 R624W	A vs. G	1,94(0.64–5.85)	0.24	R	ANCA	myeloperoxidase	1399/9934	77	0.01	
Song GG, 2013	3	PTPN22 1858C/T	T vs. C	0.482	0.216	F	Vigitio	Asian	NA	<50	>0.05	>0.05
Lee YH, 2012	9	PTPN22 1858C/T	T vs. C	1,046	0.786	R	vasculitis	Overall	1922/11505	66,8	0.002	0.634
Lee YH, 2012	6	PTPN22 1858C/T	T vs. C	1,104	0.551	R	ANCA	European		72,2	0.003	0.179
Lee YH, 2012	2	PTPN22 1858C/T	T vs. C	0.595	0.353	F	ANCA-WG	Overall		0	0.488	NA
Tang S, 2012	2	PTPN22 1858C/T	C vs T	0.727	>0.05		T1D	Asia	358/319	NA	NA	>0.05
Lea WW, 2011	1	PTPN22 1858C/T	T vs. C	1,019	0.953	NA	SLE	Africna Americna		NA	NA	NA
Lee YH, 2007	3	PTPN22 1858C/T	T vs C	0.86	0.12		IBD	Overall	3546/2779	0		
Lee YH, 2007	2	PTPN22 1858C/T	T vs. C	0.92	0.45		Psoriasis	Overall	1468/2380	0		
Lee YH, 2007	2	PTPN22 1858C/T	T vs. C	1,11	0.44		MS	Overall	998/1590	0		
Lee YH, 2007	2	PTPN22 1858C/T	T vs. C	1,18	0.16		Celiac disease	Overall	1686/2362	0		
Lee YH, 2007	2	PTPN22 1858C/T	T vs. C	1,3	0.13		Addison's disease	Overall	450/1336	50.7		
Hu LY, 2017	2	PTPN22 rs2476601	T vs C	1,323	0.532		SLE	Asian	NA	0.00	0.953	1
Hedioudje A, 2017	9			1,6	0.16	F	UC	Overall	6979/9715	32,2	0.16	0.49
Luo L, 2012	4	PTPN22 1858C/T	TT+TC vs CC	1.24(0.56,2.77)	1.00	R	AITD	Asian			>0.05	>0.05
Luo L, 2012	2	PTPN22 1858C/T	TT+TC vs CC	1.65(1.37,1.98)	0.11	R	AITD	UK			>0.05	>0.05
Luo L, 2012	2	PTPN22 1858C/T	TT+TC vs CC	1.39(0.88,2.20)	0.80	R	AITD	German			>0.05	>0.05
Luo L, 2012	7	PTPN22 1858C/T	TT+TC vs CC	1.31(0.78,2.21)	0.25	R	Hashimoto's thyroiditis	Overall			>0.05	>0.05

Lester S, 2013	5	PTPN22 1858C/T		1.31(0.90, 1.90)	0.11	R	GCA	Overall	1392/15943		
Zheng J, 2012	7	PTPN22 1858C/T	T vs. C	0.96(0.87–1.05)	0.347		UC	Overall			
Zheng J, 2012	2	PTPN22 1858C/T	T vs. C	1.11(0.85–1.44)	0.442		MS	Overall			
Zheng J, 2012	4	PTPN22 1858C/T	T vs. C	1.12(0.97–1.29)	0.125		Celiac disease	Overall			
Zheng J, 2012	3	PTPN22 1858C/T	T vs. C	1.08(0.93–1.27)	0.306		Psoriasis	Overall			
Li X, 2017		PTPN22 1858C/T		0.85(0.65-1.09)	0.20	R	Ulcerative colitis	Overall		72	0.003
Chen YF, 2012		PTPN22 1858C/T		1.08(0.92–1.28)	>0.05	R	Early-onset psoriasis	Overall		0.00	0.531
Ortiz-Fernández L, 2016	4	PTPN22 1858C/T	T vs. C	0.723(0.28-1.87)	0.504	R	BD	Overall	794/1876	69.1	0.021
Diaz-Gallo, 2011	7	PTPN22 1858C/T	T vs. C	0.98(0.85 1.15)	0.88	F	UC	Overall	5695/8766	NA	NA
Diaz-Gallo1, 2011b	8	PTPN22 1858C/T	T vs. C	0.89 (0.72 -.12)	0.36	F	SSc	Caucasian	3422/3638	17.6	.29
Diaz-Gallo1, 2011b	9	PTPN22 1858C/T	T vs. C	1.18 (0.96 - 1.44)	0.12	R	Limited cutaneous SSc	European	2546/4406	59.79	0.0109
Diaz-Gallo1, 2011b	9	PTPN22 1858C/T	T vs. C	1.09(0.94 -1.26)	0.28	F	Difused	European	1459/4406	0	0.44
Diaz-Gallo1, 2011b	9	PTPN22 1858C/T	T vs. C	1.17(0.89 - 1.55)	0.26	R	Anti-topoisomerase antibody (ATA)-positive	European	834/4126	49.9	0.04
Latiano, 2007	4	PTPN22 1858C/T	T vs C	NA	0.303	F	IBD	Overall	2275 (case+control)	<51	>0.05
Latiano, 2007	3	PTPN22 1858C/T	T vs C	NA	0.375	F	UC	Overall	1070	<53	0.99
Meng X, 2017	3	PTPN22 1858C/T	TT vs CC	1.67 (0.39, 7.06)	>0.05	F	Ankylosing Spondylitis	Overall	418/1307	0.0	0.429
de Lima SC, 2017	NA	PTPN22 1858C/T	Tvs.C	2.04 (0.65–6.44)	0.22	F	SLE	African	NA	61.77	0.1058

de Lima SC, 2017	NA	PTPN22 1858C/T	Tvs.C	1.31 (0.55–3.07	0.54	F	SLE	Asian	NA	0.000	0.9842
Curtin K, 2007	2	PTPN22 1858C/T	CC vs. CT+TT	1.20 0.87–1.65	0.31		RF-		218/1621		
Agarwal S, 2017	3	PTPN22 1858C/T	Tvs.C	0.59(0.26–1.32)	0.2	F	Vigitilo	Asian	570/674	0	0.78

CI, confidence interval; OR, odds ratio.

Table S2. Genotypic and allelic comparisons from GWAS studies not showing 95% CI.

Author, Year	NO. of studies	Gene/Variant	Comparison	OR (95% CI)	P-value	Model	Ethnicity	No. of Cases/Controls
Merkel PA, 2017	3	PTPN22 1858C/T	T vs.C	1.36	**1.77E-06**		GPA vs controls	1,556/4,723
Merkel PA,2017	3	PTPN22 1858C/T	T vs.C	1.56	**1.31E-03**		MPA vs controls	236/4,723
Merkel PA, 2017	3	PTPN22 1858C/T	T vs.C	1.10	**4.95E-01**		GPA vs.MPA	1,556/236
Merkel PA,2017	3	PTPN22 1858C/T	T vs.C	1.33	**3.19E-05**		PR3-cANCA vs. controls	1,361/4,723
Merkel PA,2017	3	PTPN22 1858C/T	T vs.C	1.64	**5.85E-06**		MPO-pANCA vs. controls	378/4,723
Merkel PA,2017	3	PTPN22 1858C/T	T vs.C	1.19	**1.40E-06**		PR3-cANCA vs MPO-pANCA	1,361/378
Bowes J, 2014	4	PTPN22 rs2476608	T vs.C	1.32	**1.49E-09**			3139/11 078)

Table S3. PRISMA Checklist.

Section/topic	#	Checklist item	Reported on page #
**TITLE**			
Title	1	Identify the report as a systematic review, meta-analysis, or both.	1
**ABSTRACT**			
Structured summary	2	Provide a structured summary including, as applicable: background; objectives; data sources; study eligibility criteria, participants, and interventions; study appraisal and synthesis methods; results; limitations; conclusions and implications of key findings; systematic review registration number.	3
**INTRODUCTION**			
Rationale	3	Describe the rationale for the review in the context of what is already known.	4-5
Objectives	4	Provide an explicit statement of questions being addressed with reference to participants, interventions, comparisons, outcomes, and study design (PICOS).	5
**METHODS**			
Protocol and registration	5	Indicate if a review protocol exists, if and where it can be accessed (e.g., Web address), and, if available, provide registration information including registration number.	N/A
Eligibility criteria	6	Specify study characteristics (e.g., PICOS, length of follow-up) and report characteristics (e.g., years considered, language, publication status) used as criteria for eligibility, giving rationale.	5-8 Figure 1
Information sources	7	Describe all information sources (e.g., databases with dates of coverage, contact with study authors to identify additional studies) in the search and date last searched.	6
Search	8	Present full electronic search strategy for at least one database, including any limits used, such that it could be repeated.	6-8 Figure 1
Study selection	9	State the process for selecting studies (i.e., screening, eligibility, included in systematic review, and, if applicable, included in the meta-analysis).	6-8 Figure 1
Data collection process	10	Describe method of data extraction from reports (e.g., piloted forms, independently, in duplicate) and any processes for obtaining and confirming data from investigators.	6, 8

Data items	11	List and define all variables for which data were sought (e.g., PICOS, funding sources) and any assumptions and simplifications made.	6, 8
Risk of bias in individual studies	12	Describe methods used for assessing risk of bias of individual studies (including specification of whether this was done at the study or outcome level), and how this information is to be used in any data synthesis.	N/A
Summary measures	13	State the principal summary measures (e.g., risk ratio, difference in means).	6, 8-9
Synthesis of results	14	Describe the methods of handling data and combining results of studies, if done, including measures of consistency (e.g., I^2) for each meta-analysis.	8-9

Section/topic	#	Checklist item	Reported on page #
Risk of bias across studies	15	Specify any assessment of risk of bias that may affect the cumulative evidence (e.g., publication bias, selective reporting within studies).	N/A
Additional analyses	16	Describe methods of additional analyses (e.g., sensitivity or subgroup analyses, meta-regression), if done, indicating which were pre-specified.	N/A
**RESULTS**			
Study selection	17	Give numbers of studies screened, assessed for eligibility, and included in the review, with reasons for exclusions at each stage, ideally with a flow diagram.	10
Study characteristics	18	For each study, present characteristics for which data were extracted (e.g., study size, PICOS, follow-up period) and provide the citations.	10
Risk of bias within studies	19	Present data on risk of bias of each study and, if available, any outcome level assessment (see item 12).	N/A
Results of individual studies	20	For all outcomes considered (benefits or harms), present, for each study: (a) simple summary data for each intervention group (b) effect estimates and confidence intervals, ideally with a forest plot.	10-25<br>Table 1-9
Synthesis of results	21	Present results of each meta-analysis done, including confidence intervals and measures of consistency.	10-25<br>Table 1-9
Risk of bias across studies	22	Present results of any assessment of risk of bias across studies (see Item 15).	N/A
Additional analysis	23	Give results of additional analyses, if done (e.g., sensitivity or subgroup analyses, meta-regression [see Item 16]).	N/A
**DISCUSSION**			

Summary of evidence	24	Summarize the main findings including the strength of evidence for each main outcome; consider their relevance to key groups (e.g., healthcare providers, users, and policy makers).	25-29
Limitations	25	Discuss limitations at study and outcome level (e.g., risk of bias), and at review-level (e.g., incomplete retrieval of identified research, reporting bias).	29-30
Conclusions	26	Provide a general interpretation of the results in the context of other evidence, and implications for future research.	30
**FUNDING**			
Funding	27	Describe sources of funding for the systematic review and other support (e.g., supply of data); role of funders for the systematic review.	31

From: Moher D, Liberati A, Tetzlaff J, Altman DG, The PRISMA Group (2009). Preferred Reporting Items for Systematic Reviews and Meta-Analyses: The PRISMA Statement. PLoS Med 6(7): e1000097. doi:10.1371/journal.pmed1000097.

Supplemental references

1. Nabi, G.; Akhter, N.; Wahid, M.; Bhatia, K.; Mandal, K. R.; Dar, S. A.; Jawed, A.; Haque, S., Meta-analysis reveals PTPN22 1858C/T polymorphism confers susceptibility to rheumatoid arthritis in Caucasian but not in Asian population. Autoimmunity. 2016, 49 (3), 197-210, doi: 10.3109/08916934.2015.1134514.

2 Elshazli, R.; Settin, A., Association of PTPN22 rs2476601 and STAT4 rs7574865polymorphisms with rheumatoid arthritis: A meta-analysis update. Immunobiology2015, 220 (8), 1012–1024, doi: 10.1016/j.imbio.2015.04.003.

3 Tang, S.; Peng, W.; Wang, C.; Tang, H.; & Zhang, Q., Association of the PTPN22 gene (+1858C/T, −1123G/C) polymorphisms with type 1 diabetes mellitus: A systematic review and meta-analysis. Diabetes Res. Clin. Pract.2012, 97 (3), 446–452, doi: 10.1016/j.diabres.2012.04.011.

4 Song, G. G.; Bae, S.-C.; Kim, J.-H.; Lee, Y. H., The PTPN22 C1858T polymorphism and rheumatoid arthritis: a meta-analysis. Rheumatol. Int.2013, 33 (8), 1991–9, doi:10.1007/s00296-013-2679-2.

5 Zheng, J.; Ibrahim, S.; Petersen, F.; Yu, X., Meta-analysis reveals an association of PTPN22 C1858T with autoimmune diseases, which depends on the localization of the affected tissue. Genes Immun.2012, 13 (8), 641–52, doi: 10.1038/gene.2012.46.

6 Lee, Y. H.; Choi, S.-J.; Ji, J.-D.; Choi, S.-J.; Ji, J.-D.; Song, G.-G., The association between the PTPN22 C1858T polymorphism and rheumatoid arthritis: a meta-analysis update. Mol. Biol. Rep.2012, 39 (4), 3453–60, doi: 10.1007/s11033-011-1117-3.

7 Jiang, Y. ; Zhang, R.; Zheng, J. ; Liu, P. ; Tang, G.; Lv, H. ; et al., Meta-Analysis of 125 Rheumatoid Arthritis-Related Single Nucleotide Polymorphisms Studied in the Past Two Decades. PLoS One2012, 7 (12), e51571, doi: 10.1371/journal.pone.0051571 .

8 Ramirez, M.; Quintana, G.; Diaz-Gallo, L. M.; Caminos, J.; Garces, M.; Cepeda, L.; et al., The PTPN22 C1858T variant as a risk factor for rheumatoid arthritis and systemic lupus erythematosus but not for systemic sclerosis in the Colombian population. Clin. Exp. Rheumatol. 2012, 30 (4), 520-4, PMID: 22704547.

9 Nong, L. M.; Ren, K. W.; Xu, N. W.; Zhou, D., 1858 C/T polymorphism of the protein tyrosine phosphatase nonreceptor 22 gene and rheumatoid arthritis risk in europeans: a meta-analysis. Arch. Med. Res.2011, 42 (8), 698-702, doi: 10.1016/j.arcmed.2011.12.001.

10 Totaro, M. C.; Tolusso, B.; Napolioni, V.; Faustini, F.; Canestri, S.; Mannocci, A.; Gremese, E.; Bosello, S. L.; Alivernini, S.; Ferraccioli, G., PTPN22 1858C.T Polymorphism Distribution in Europe and Association with Rheumatoid Arthritis: Case-Control Study and Meta-Analysis. Plos One2011, 6 (9), e24292, doi: 10.1371/journal.pone.0024292.

11 Plant, D.; Flynn, E.; Mbarek, H.; Dieudé, P.; Cornelis, F.; Ärlestig, L.; et al., Investigation of potential non-HLA rheumatoid arthritis susceptibility loci in a European cohort increases the

evidence for nine markers. Ann. Rheum. Dis.2010, 69 (8), 1548–53, doi: 10.1136/ard.2009.121020.

12 Curtin, K.; Wong, J.; Allen-Brady, K.; Camp, N. J., Meta-genetic association of rheumatoid arthritis and PTPN22 using PedGenie 2.1. BMC Proc.2007, 1(Suppl 1), S12, PMCID: PMC2367587.

13 Lee, Y. H.; Rho, Y. H.; Choi, S. J.; Ji, J. D.; Song, G. G.; Nath, S. K.; Harley, J. B., The PTPN22 C1858T functional polymorphism and autoimmune diseases—a meta-analysis. Rheumatology (Oxford)2007, 46 (1), 49–56, doi: 10.1093/rheumatology/kel170.

14 Di, Y.; Zhong, S.; Wu, L.; Li, Y.; Sun, N., The Association between PTPN22 Genetic Polymorphism and Juvenile Idiopathic Arthritis (JIA) Susceptibility: An Updated Meta-Analysis. Iran J. Public Health2015, 44 (9), 1169-75, PMCID: PMC4645773.

15 Kaalla, J. M.; Broadaway, K. A.; Rohani-Pichavant, M.; Conneely, K. N.; Whiting, A.; Ponder, L.; Okou, D. T.; Angeles-Han, S.; Rouster-Stevens, K.; Brown, M. R.; Vogler, L. B.; Jorde, L. B.; Bohnsack, J. F.; Epstein, M. P.; Prahalad, S., Meta-analysis confirms association between TNFA-G238A variant and JIA, and between PTPN22-C1858T variant and oligoarticular, RF-polyarticular and RF-positive polyarticular JIA. Pediatr. Rheumatol. Online J. 2013, 11 (1). 40, doi: 10.1186/1546-0096-11-40.

16 Lee, Y. H.; Bae, S.-C.; Song, G. G., The association between the functional PTPN22 1858 C/T and MIF −173 C/G polymorphisms and juvenile idiopathic arthritis: a meta-analysis. Inflamm. Res.2012, 61(5), 411–5, doi: 10.1007/s00011-012-0447-5.

17 Hu, L. Y.; Cheng, Z.; Zhang, B.; Yin, Q.; Zhu, X.-W.; Zhao, P.-P.; Han, M.-Y.; Wang, X.-B.; Zheng, H.-F., Associations between PTPN22 and TLR9 polymorphisms and systemic lupus erythematosus: a comprehensive meta-analysis. Arch. Dermatol. Res.2017, 309 (6), 461-477, doi: 10.1007/s00403-017-1745-0.

18 de Lima, S. C.; Adelino J. E.; Crovella S.; de Azevedo Silva, J.; Sandrin-Garcia P., PTPN22 1858C > T polymorphism and susceptibility to systemic lupus erythematosus: a meta-analysis update. Autoimmunity. 2017, 50 (7), 428-434, doi: 10.1080/08916934.2017.1385774.

19 Shi, L.; Wei, Y.; Xun, W.; Han, D., Meta-Analysis of the Correlation Between PTPN22 Gene Polymorphisms and Susceptibility to Systemic Lupus Erythematosus. Asia Pac. J. Public Health 2013 25 (4 Suppl), 22S–29S, doi: 10.1177/1010539513496268.

20 Lea, W.W.; Lee, Y.H., The association between the PTPN22 C1858T polymorphism and systemic lupus erythematosus: a meta-analysis update. Lupus. 2011, 20, 51–57, doi: 10.1177/0961203310381774.

21 Rahmattulla, C.; Mooyaart, A. L.; van Hooven, D.; Schoones, J. W.; Bruijn, J. A.; Dekkers, O. M.; Bajema, I. M., European Vasculitis Genetics Consortium, Genetic variants in ANCA-associated vasculitis: a meta-analysis. Ann. Rheum. Dis.2015, 75 (9), 1687-92, doi: 10.1136/annrheumdis-2015-207601.

22 Cao, Y.; Liu, K.; Tian, Z.; Hogan, S. L.; Yang, J.; Poulton, C. J.; Falk, R.J.; Li, W., PTPN22 R620W polymorphism and ANCA disease risk in white populations: a metaanalysis. J. Rheumatol.2015, 42 (2), 292-9, doi: 10.3899/jrheum.131430.

23 Lee, Y. H.; Choi, S.-J.; Ji, J.-D.; Song, G.-G., The protein tyrosine phosphatase nonreceptor 22 C1858T polymorphism and vasculitis: a meta-analysis. Mol. Biol. Rep.2012, 39 (8), 8505–11, doi: 10.1007/s11033-012-1705-x. vascultis.

24 Lester, S.; Hewitt, A.; Bradbury, L.; De Smit, E.; Harrison, A.; Jones, G.; et al., PTPN22 rs2476601 and Susceptibility to Biopsy Proven Giant Cell Arteritis (GCA) in an Australian Sample. Ann. Rheum. Dis. 2013, 72, 1882-6.

25 Diaz-Gallo, L. M.; Gourh, P.; Broen, J.; Simeon, C.; Fonollosa, V.; Ortego-Centeno, N.; et al., Analysis of the influence of PTPN22 gene polymorphisms in systemic sclerosis. Ann. Rheum. Dis.2011, 70 (3), 454–62, doi: 10.1136/ard.2010.130138.

26 Dieudé, P.; Guedj, M.; Wipff, J.; Avouac, J.; Hachulla, E.; Diot, E.; Granel, B.; et al., The PTPN22 620W Allele Confers Susceptibility to Systemic Sclerosis Findings of a Large Case–Control Study of European Caucasians and a Meta-Analysis. Arthritis Rheum.2008, 58 (7), 2183–8, doi: 10.1002/art.23601.

27 Chen, Y.-F.; Chang, J.-S., PTPN22 C1858T and the risk of psoriasis: a meta-analysis. Mol. Biol. Rep.2012, 39 (8), 7861–70, doi: 10.1007/s11033-012-1630-z.

28 Meng, X.; Wang, W.; Liu, Y.; Ma, X.; Zhang, Q.; Li, C.; Li, C.; Ren, L.; Association Between Protein Tyrosine Phosphatase Non-Receptor Type 22 (PTPN22) Polymorphisms and Risk of Ankylosing Spondylitis: A Meta-analysis. Med. Sci. Monit, 2017, 23, 2619-24, PMCID: PMC5461884.

29 Agarwal, S.; Changotra, H., Association of protein tyrosine phosphatase, non-receptor type 22 +1858C→T polymorphism and susceptibility to vitiligo: Systematic review and meta-analysis. Indian J. Dermatol. Venereol. Leprol.2017, 83 (2), 183-189, doi: 10.4103/0378-6323.199422.

30 Song, G. G.; Kim, J.-H.; Lee, Y. H., The CTLA-4 +49 A/G, CT60 A/G and PTPN22 1858 C/T polymorphisms and susceptibility to vitiligo: a meta-analysis. Mol. Biol. Rep. 2013, 40 (4), 2985-93, doi: 10.1007/s11033-012-2370-9.
31 Hedjoudje, A.; Cheurfa, C.; Briquez, C.; Zhang, A.; Koch, S.; Vuittonr, L.; s2476601 polymorphism in PTPN22 is associated with Crohn's disease but not with ulcerative colitis: a meta-analysis of 16,838 cases and 13,356 controls. Ann. Gastroenterol.2017, 30 (2), 197-208, doi: 10.20524/aog.2017.0121.
32 Li, X.; Niu, M.; Yang, H.; Zhou, F.; Xi, Z., Protein tyrosine phosphatase nonreceptor type 22 (PTPN22) gene R620W polymorphism is associated with inflammatory bowel disease risk. Int. J. Clin. Exp. Med.2017, 10 (7), 9857-63.
33 Diaz-Gallo, L. M.; Espino-Paisan, L.; Fransen, K.; Gomez-Garcıa, M.; van Sommeren, S.; Cardena, C.; et al., Differential Association of Two PTPN22 Coding Variants with Crohn's Disease and Ulcerative Colitis. Inflamm. Bowel Dis.2011, 17 (11), 2287-88, doi: 10.1002/ibd.21630.
34 Latiano, A.; Palmieri, O.; Valvano, M. R.; Bossa, F.; Latiano, T.; Corritore, G.; et al., Evaluating the Role of the Genetic Variations of PTPN22, NFkB1, and FcGRIIIA Genes in Inflammatory Bowel Disease: A Meta-analysis. Inflamm. Bowel Dis.2007, 13 (10), 1212–9, doi: 10.1002/ibd.20185.
35 Xiong, X.; Xiang, M.; Cheng, X.; Huang, Y., PTPN22 R620W Polymorphism is Associated with Myasthenia Gravis Risk: A Systematic Review and Meta-Analysis. Med. Sci. Monit,2015 21, 2567-71, doi: 10.12659/MSM.894307.
36 Provenzano, C.; Ricciardi, R.; Scuderi, F.; Maiuri, M. T.; Maestri, M.; La Carpia, F.; et al., PTPN22 and myasthenia gravis: Replication in an Italian population and meta-analysis of literature data. Neuromuscul. Disord.2012 22 (2), 131–8, doi: 10.1016/j.nmd.2011.09.003.
37 Luo, L.; B, Cai.; Liu, F.; Hu, X.; Wang, L., Association of Protein Tyrosine Phosphatase Nonreceptor 22 (PTPN22) C1858T gene polymorphism with susceptibility to autoimmune thyroid diseases: a meta-analysis. Endocr. J.2012, 59 (5), 439-45, PMID: 22374328
38 Skinningsrud ,B , Husebye ES, Gervin K, Lovaas K, Blomhoff A, Wolff A B, et al. Mutation screening of PTPN22: association of the 1858T-allele with Addison's disease. European Journal of Human Genetics (2008) 16, 977–982.
39 Roycroft, M.; Fichna, M.; McDonald, D.; Owen, K.; Zurawek, M.; Gryczynska, M.; et al., The tryptophan 620 allele of the lymphoid tyrosine phosphatase (PTPN22) gene predisposes to autoimmune Addison's disease. Clin. Endocrinol. (Oxf).2009, 70, 358–362, doi: 10.1111/j.1365-2265.2008.03380.x.
40 Pabalan, N.; Jarjanazi, H.; Christofolini, D. M.; Bianco, B.; Barbosa, C. P.; Association of the protein tyrosine phosphatase non-receptor 22 polymorphism (PTPN22) with endometriosis: a meta-analysis. Einstein (Sao Paulo)2017, 15 (1), 105-11, doi: 10.1590/S1679-45082017RW3827.
41 Ramu, D.; Perumal, V.; Paul, S. F. D., Association of common type 1 and type 2 diabetes gene variants (PTPN22, INS, and TCF7L2) with Latent Autoimmune Diabetes in Adults (LADA): a meta-analysis. J. Diabetes2018, doi:10.1111/1753-0407.12879.
42 Dong, F.; Yang, G.; Pan, H. W.; Huang, W. H.; Jing, L. P.; Liang, W. K.; Zhang, N.; et al., The association of PTPN22 rs2476601 polymorphism and CTLA-4 rs231775 polymorphism with LADA risks: a systematic review and meta-analysis. Acta. Diabetol.2014, 51 (5), 691-703, doi: 10.1007/s00592-014-0613-z.
43 Xuan, C.; Lun, L. M.; Zhao, J. X.; Wang, H. W.; Zhu, B. Z.; Yu, S.; Liu, Z.; He, G. W., PTPN22 gene polymorphism (C1858T) is associated with susceptibility to type 1 diabetes: a meta-analysis of 19,495 cases and 25,341 controls. Ann. Hum. Genet.2013, 77 (3), 191-203, doi:10.1111/ahg.12016.
44 Wang, X. F.; Chen, Z. X.; Shao, Y. C.; Ma, Y. S.; Zhang, F.; Zhang, L.; et al., Population-based and family-based studies on the protein tyrosine phosphatase non-receptor 22 gene polymorphism and type 1 diabetes: A meta-analysis. Gene2013, 517 (2), 191–196. doi: 10.1016/j.gene.2012.12.076.
45 Tang, G. P. ; Hu, L. ; Zhang, Q. H., [PTPN22 1858C/T polymorphism is associated with rheumatoid arthritis susceptibility in Caucasian population: a meta-analysis]. Zhejiang Da XueXue Bao Yi Xue Ban. 2014, 43 (4), 466-73, PMID: 25187463.
46 Peng, H.; Zhou, M.; Xu, W. D.; Xu, K.; Zhai, Y.; Li, R.; et al., Association of PTPN22 C1858T Polymorphism and Type 1 Diabetes: A Meta-analysis. Immunol. Invest.2012, 41 (5), 484–496, doi: 10.3109/08820139.2012.664226.
Meta-analyses of GWAS
47 Bowes, J.; Loehr, S.; Budu-Aggrey, A.; Uebe, S.; Bruce, I. N.; Feletar, M.; Marzo-Ortega, H.; et al., PTPN22 is associated with susceptibility to psoriatic arthritis but not psoriasis: evidence for a further PsA-specific risk locus. Ann. Rheum. Dis.2015, 74 (10), 1882–5, doi: 10.1136/annrheumdis-2014-207187 .

48 Gregersen, P. K.; Kosoy, R.; Lee, A. T.; Lamb, J.; Sussman, J.; McKee, D.; et al., Risk for Myasthenia Gravis maps to 151Pro→ Ala change in TNIP1 and to HLA-B*08. Ann. Neurol. 2012, 72 (6), 927–935, doi: 10.1002/ana.23691.
49 Thompson, S. D.; Sudman, M.; Ramos, P. S.; Marion, M. C.; Ryan, M.; Tsoras, M.; et al., The susceptibility loci Juvenile Idiopathic Arthritis shares with other autoimmune diseases extend to PTPN2, COG6 and ANGPT1. Arthritis Rheum.2010, 62 (11), 3265–76, doi: 10.1002/art.27688.
50 Coenen, MJ.; Trynka, G.; Heskamp, S.; Franke, B.; van Diemen, C. C.; Smolonska, J.; et al., Common and different genetic background for rheumatoid arthritis and coeliac disease. Hum. Mol. Genet.2009, 18 (21), 4195–4203, doi: 10.1093/hmg/ddp365.
51 Merkel, P. A.; Xie, G.; Monach, P. A.; Ji, X.; Ciavatta, D. J.; Byun, J.; et al., Identification of Functional and Expression Polymorphisms Associated With Risk for Antineutrophil Cytoplasmic Autoantibody–Associated Vasculitis. Arthritis Rheumatol. 2017, 69 (5), 1054–1066, doi: 10.1002/art.40034.
52 Törn, C.; Hadley, D.; Lee, H.-S.; Hagopian, W.; Lernmark, Å.; Simel, O.; et al., Role of Type 1 Diabetes–Associated SNPs on Risk of Autoantibody Positivity in the TEDDY Study. Diabetes2015, 64 (5), 1818–29, doi: 10.2337/db14-1497.
53 Serrano, A.; Márquez, A.; Mackie, S. L.; Carmona, F. D.; Solans, R.; Miranda-Filloy, J. A.; et al., Identification of the PTPN22 functional variant R620W as susceptibility genetic factor for giant cell arteritis. Ann. Rheum. Dis.2013, 72 (11), 1882–1886, doi: 10.1136/annrheumdis-2013-203641.
